# Induction of *Aspergillus fumigatus* zinc cluster transcription factor OdrA/Mdu2 provides combined cellular responses for oxidative stress protection and multiple antifungal drug resistance

**DOI:** 10.1128/mbio.02628-23

**Published:** 2023-11-20

**Authors:** Christoph Sasse, Emmanouil Bastakis, Fruzsina Bakti, Annalena M. Höfer, Isabella Zangl, Christoph Schüller, Anna M. Köhler, Jennifer Gerke, Sven Krappmann, Florian Finkernagel, Rebekka Harting, Joseph Strauss, Kai Heimel, Gerhard H. Braus

**Affiliations:** 1 Department of Molecular Microbiology and Genetics and Göttingen Center for Molecular Biosciences (GZMB), Institute of Microbiology and Genetics, University of Göttingen, Göttingen, Germany; 2 Department of Applied Genetics and Cell Biology, Institute of Microbial Genetics, University of Natural Resources and Life Sciences, Vienna (BOKU), Campus, Tulln, Austria; 3 Core Facility Bioactive Molecules–Screening and Analysis, University of Natural Resources and Life Sciences, Vienna (BOKU), Austria; 4 Institute of Microbiology–Clinical Microbiology, Immunology and Hygiene, University Hospital Erlangen and Friedrich-Alexander University Erlangen-Nürnberg, Erlangen, Germany; 5 Center for Infection Research (ECI) and Medical Immunology Campus Erlangen (MICE), Erlangen, Germany; 6 Center for Tumor Biology and Immunology, Core Facility Bioinformatics, Philipps University, Marburg, Germany; 7 Department of Microbial Cell Biology, Institute of Microbiology and Genetics, Göttingen Center for Molecular Biosciences (GZMB), University of Göttingen, Göttingen, Germany; IMBB-FORTH, Heraklion, Greece

**Keywords:** *Aspergillus fumigatus*, oxidative stress, mdr1, zinc cluster transcription factors, drug tolerance, atrR, OdrA/Mdu2

## Abstract

Zinc cluster transcription factors (Zcfs) encoding *zcf* genes are exclusive for fungi and required for multiple cellular processes including metabolism and development. Genome-wide screening of 228 zinc cluster transcription factor encoding genes by overexpression in *Aspergillus fumigatus* revealed 11 genes which provided increased tolerance to the broadly applied azole voriconazole or to the polyene amphotericin B or to both. These include four oxidative stress and drug resistance genes (*odrA-D*) genes encoding factors, which provide broad cellular stress protection. Thereby, the corresponding fungal OdrA/Mdu2- and AtrR/OdrD-dependent genetic networks are interconnected. OdrA/Mdu2 activates *atrR*/*odrD* transcription by direct binding to the promoter, whereas AtrR/OdrD functions as repressor of *odrA*/*mdu2* expression. *odrA*/*mdu2* overexpression provides combined resistance to amphotericin B, voriconazole, itraconazole, and reactive oxygen species generated by menadione. OdrA/Mdu2-mediated itraconazole resistance is evoked by direct regulation of the transporter encoding gene *mdr1*. Oxidative stress-inducing substances like amphotericin B and menadione promote OdrA/Mdu2 accumulation in the nucleus to regulate stress response genes like *mdr1* and the putative glutathione-S-transferase encoding gene *gstD*. The expression levels and external stress conditions fostering nuclear accumulation of OdrA/Mdu2 determine the regulation of the target genes. Hence, OdrA/Mdu2 provides a combined adaptation strategy for survival in nature or within a potential host, where this fungus represents the most common agent for human mold pneumonia worldwide. The OdrA/Mdu2 controlled genetic network highlights the tight connection between oxidative stress response and antifungal drug adaption to secure *A. fumigatus* survival in various hostile environments.

**IMPORTANCE:**

An overexpression screen of 228 zinc cluster transcription factor encoding genes of *A. fumigatus* revealed 11 genes conferring increased tolerance to antifungal drugs. Out of these, four oxidative stress and drug tolerance transcription factor encoding *odr* genes increased tolerance to oxidative stress and antifungal drugs when overexpressed. This supports a correlation between oxidative stress response and antifungal drug tolerance in *A. fumigatus*. OdrA/Mdu2 is required for the cross-tolerance between azoles, polyenes, and oxidative stress and activates genes for detoxification. Under oxidative stress conditions or when overexpressed, OdrA/Mdu2 accumulates in the nucleus and activates detoxifying genes by direct binding at their promoters, as we describe with the *mdr1* gene encoding an itraconazole specific efflux pump. Finally, this work gives new insights about drug and stress resistance in the opportunistic pathogenic fungus *A. fumigatus*.

## INTRODUCTION

*Aspergillus fumigatus* is one of the most important human pathogenic fungi and causes severe invasive aspergillosis in immunocompromised patients with mortality rates from 38% to 85% (1–3). The sustained treatment of patients with azoles as well as their usage in agriculture increase the frequency of resistant *A. fumigatus* strains (4–6).

Polyenes are rarely used in agriculture but are naturally produced by *Streptomycetes* species in competition with fungi in soil. Therefore, *A. fumigatus* requires and relies on effective systems to ensure survival in the presence of various groups of antifungal compounds (7, 8). In most cases, azole resistance of *A. fumigatus* has been related to the fungal drug target gene *cyp51A* encoding the lanosterol 14-α demethylase important for ergosterol biosynthesis. However, several strains have been isolated in recent years displaying high tolerance to azoles, for which neither mutations nor overexpression of the *cyp51A* gene was detectable (9, 10). Activation of the oxidative stress response or upregulation of genes encoding ABC and MFS transporters appears to be relevant resistance mechanisms for cellular detoxification beside *cyp51A* mutations (11–13). By these mechanisms, most of the observed resistance phenotypes can be explained. The molecular relationship between drug resistance and oxidative stress response in *A. fumigatus* is yet elusive. A correlation between drug and oxidative stress adaption was described for the Gβ-like protein CpcB, which also affects fungal adhesion and development (14–17). Artificial hyperactive Yap1, which is a key regulator for oxidative stress response, promotes decreased susceptibility to reactive oxygen species (ROS) and voriconazole, whereas overexpression of the *yap1* wild-type gene is not sufficient for elevated voriconazole resistance (18, 19). It is currently unclear if additional transcription factors regulate the combined oxidative stress and antifungal drug tolerance in *A. fumigatus*.

Another common mechanism for the adaption to azoles of *A. fumigatus* is related to the increased expression of ABC drug efflux transporters encoding genes like *mdr1* and *abcB*/*cdr1B* (20, 21). The C2H2 transcription factor SltA induces *mdr1* expression and the ABC-transporter regulating transcription factor (AtrR)/OdrD induces *abcB* expression and corresponding efflux transporters (22–24). AtrR/OdrD belongs to the large group of zinc cluster transcription factors (zcf’s), which are exclusive for fungi and are required in different cellular processes as well as for drug resistance like Mrr1 and Mrr2 in *Candida albicans* (25–28). The genome of *A. fumigatus* comprises an expanded arsenal of more than 220 genes predicted to encode zinc cluster proteins, in comparison to the reduced set of Zcf encoding genes in *C. albicans* (82 genes) and *Saccharomyces cerevisiae* (55 genes) (27, 28). In contrast to these two yeasts (27, 28), it is largely unknown how this group of regulators is involved in azole tolerance in *A. fumigatus*. So far, only AtrR/OdrD has been characterized in this respect (22, 24).

Mechanisms conferring resistance to a group of antifungal drugs greatly reduce treatment options and increase mortality rates of patients with aspergillosis (29). Cross-resistance to different azoles is often found in *A. fumigatus*, whereas cross-resistance to azoles and polyenes seems to be not very common and is still poorly understood (30, 31). The transcriptional regulators NctA and NctB were identified as negative regulators for the combined resistance against polyenes and azoles (32).

We examined the effect of induced overexpression of 228 *zcf* genes on drug resistance of *A. fumigatus*. Eleven candidates of all tested genes decreased the susceptibility to antifungals, corroborating the importance of this group of genes for antifungal drug response. Four of these 11 genes increased oxidative stress and drug resistance (*odr*) when overexpressed. These *odrA-D* genes include *atrR* (*odrD*) and *mdu2* (*odrA*), which were previously found in correlation with voriconazole resistance (11, 22, 24). Our detailed analysis of OdrA/Mdu2 revealed that increased levels and nuclear accumulation provide protection to amphotericin B, itraconazole, and oxidative stress in addition to the known effects on voriconazole resistance (11). The OdrA/Mdu2-induced broad-spectrum responses range from specific drug efflux improvement like *mdr1* expression to more general cellular protection measures. Increased nuclear accumulation is triggered by amphotericin B/menadione providing novel insights into the mechanism of the OdrA/Mdu2-mediated response.

## RESULTS

### High expression levels of *odrA/mdu2*, *odrC*, and *atrR*/*odrD* promote cross-tolerance to amphotericin B and voriconazole

Our current knowledge about *zcf* genes in *A. fumigatus* is limited, as only few of the corresponding genes have yet been characterized in detail (24, 26, 33–39). Combined evidence from genome-wide gene deletion analyses and from artificially activated zinc cluster transcription factors in *A. fumigatus* and *C. albicans*, respectively, suggests an important role of these regulators for drug tolerance (28, 32). The function of the large group of *A. fumigatus* zinc cluster transcription factors was approached by individually overexpressing all genes encoding a protein that contains a predicted C_6_-Zn_2_ DNA-binding domain to investigate a potential connection between these regulators and antifungal drug resistance. Using the zinc cluster DNA binding domain (accession no. cd00067, National Center for Biotechnology Information [NCBI] [40]; accession no. IPR036864, CADRE/EnsemblFungi [41]) as proxy for blastp search against the *A. fumigatus* proteome (Af293) complemented by the Fungal and Oomycete Genomic Resources Database (FungiDB) (42), we identified 228 candidate *zcf* genes (Table S1).

**Fig 1:**
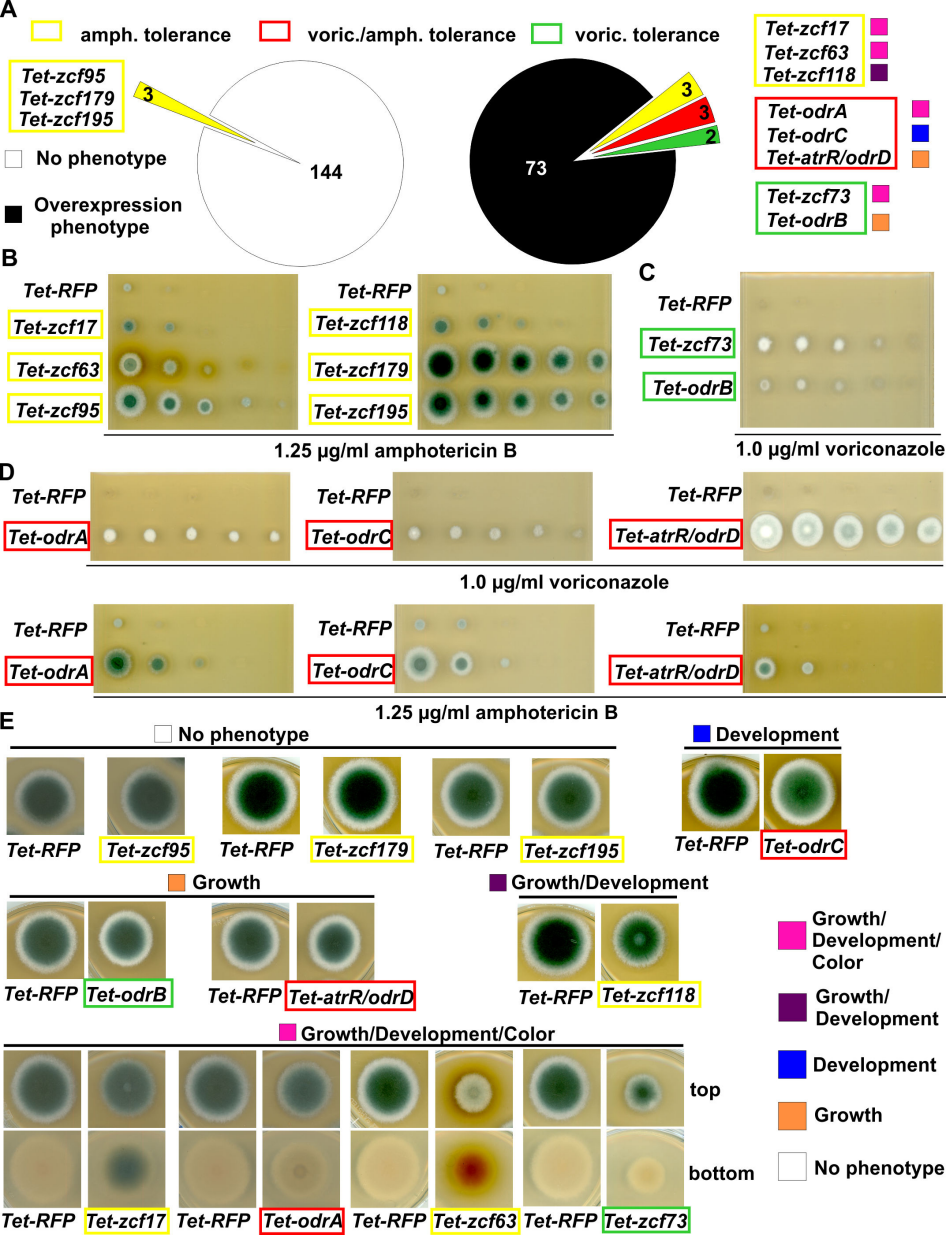
Identification of zinc cluster transcription factors (*zcf*s) encoding genes involved in drug tolerance. (**A**) Pie charts depict the number of strains showing tolerance without (left) or with an additional overexpression phenotype (right) based on the phenotype classification of Fig. S2. (**B–D**) Dilution spot tests of the different strains leading to an increased tolerance to amphotericin B (yellow) (**B**), voriconazole (green) (**C**), or both drugs (red) (**D**). The *Tet-RFP* strain containing *mCherry* under the *TetOn*-promoter instead of a *zcf* gene was used as control. Strains were diluted in 1 of 10 steps starting with 1.5 × 10^5^ spores. Strains were grown for 3 days at 37°C. (**E**) Overexpression phenotypes without additional drugs. Approximately 2,000 spores were spotted on minimal medium with 50-µg/mL doxycycline to induce the *TetOn* promoter. Plates were incubated for 3 days at 37°C. The color legend of the filled squares indicates the overexpression phenotype as mentioned in Fig. S2. The colored frames stand for the corresponding drug tolerance.

Overexpression strains were generated by homologous recombination of individual constructs (*TetOn*-promoter with gene of interest) into a ∆*pyroA* strain at the *pyroA* endogenous genomic locus (Fig. S1 and S2). This results into the integration of an additional copy of the gene of interest, ensuring increased expression levels compared to the wild type. A strain expressing *RFP* (red fluorescence protein) under the control of the *Tet* promoter (*Tet-RFP*) was tested via microscopy and was used as control for all overexpression experiments (Fig. S2C). Elevated expression levels of 15 *zcf* genes under control of the *TetOn* system were verified by quantitative PCR (qPCR). The 15 overexpression strains include those with increased antifungal drug tolerance in the presence of doxycycline (Fig. S4). Overexpression of 81 candidate genes led to phenotypical alterations under asexual conditions (Fig. S3 and S5; Table S2). Eleven candidates were identified whose overexpression affected resistance to amphotericin B (six genes), voriconazole (two genes), or both drugs (three genes) (Fig. 1). Nine of these *zcf* genes are yet uncharacterized (Table S1), and only *zcf46*/*odrA*/*mdu2* and *atrR*/*odrD*/*zcf228* have been described in previous works with regard to drug resistance, validating our assay setup (11, 22, 24). *zcf46*/*odrA* encodes the Mdu2 protein, which was named mitochondrial dynamics upregulated gene, since expression is increased in *A. fumigatus* strains deficient in mitochondrial fission and fusion (11). This regulator shows also a weak homology to Mrr2 from *Candida albicans*, which promotes fluconazole resistance by regulating *CDR1* (28). The 11 candidates were further analyzed for fitness defects in liquid culture (vegetative growth) when overexpressed. It was observed that some strains displaying a phenotype during asexual conditions have no effect on fitness under vegetative growth (*Tet-zcf17*, *Tet-odrA*, *Tet-zcf118*, and *Tet-odrB*), whereas one shows a vice versa effect (*Tet-zcf179*) (Table S3; Fig. S6). These data demonstrate that phenotypical effects in correlation with drug resistance often depend on growth or developmental conditions.

Our study revealed that *atrR*/*odrD* and *odrA*/*mdu2* are both part of the *odr* subgroup of *zcf* genes encoding oxidative stress and drug resistance factors promoting cross-tolerance between antifungals and oxidative stress when overexpressed.

Overexpression of *zcf95*, *zcf179*, and *zcf195* did not lead to an obvious phenotype in the absence of drugs but exclusively increased resistance to amphotericin B. Overexpression of *odrA/mdu2*, *zcf219/odrC*, or *atrR/odrD* diminished the susceptibility to amphotericin B and voriconazole, whereas overexpression of *zcf17*, *zcf63*, and *zcf118* only increased the resistance to amphotericin B (Fig. 1A through D). Overexpression of *zcf73* and *zcf215*/*odrB* promoted resistance only to voriconazole. The increased resistance of these eight overexpressing strains is correlated with additional phenotypes in the absence of drugs compared to the *Tet-RFP* control strain (Table S1; Fig. 1E).

These data imply that overexpression of different zinc cluster transcription factors can result in a resistance to single groups of antifungal drugs like azoles or polyenes, as well as to cross-resistance to different groups of antifungal drugs in *A. fumigatus*. In comparison to amphotericin B, voriconazole resistance appears to be frequently correlated with phenotypical defects.

### Overexpression of *odrA/mdu2* or *atrR/odrD* mediates cross-resistance to amphotericin B, itraconazole, voriconazole, and menadione

**Fig 2:**
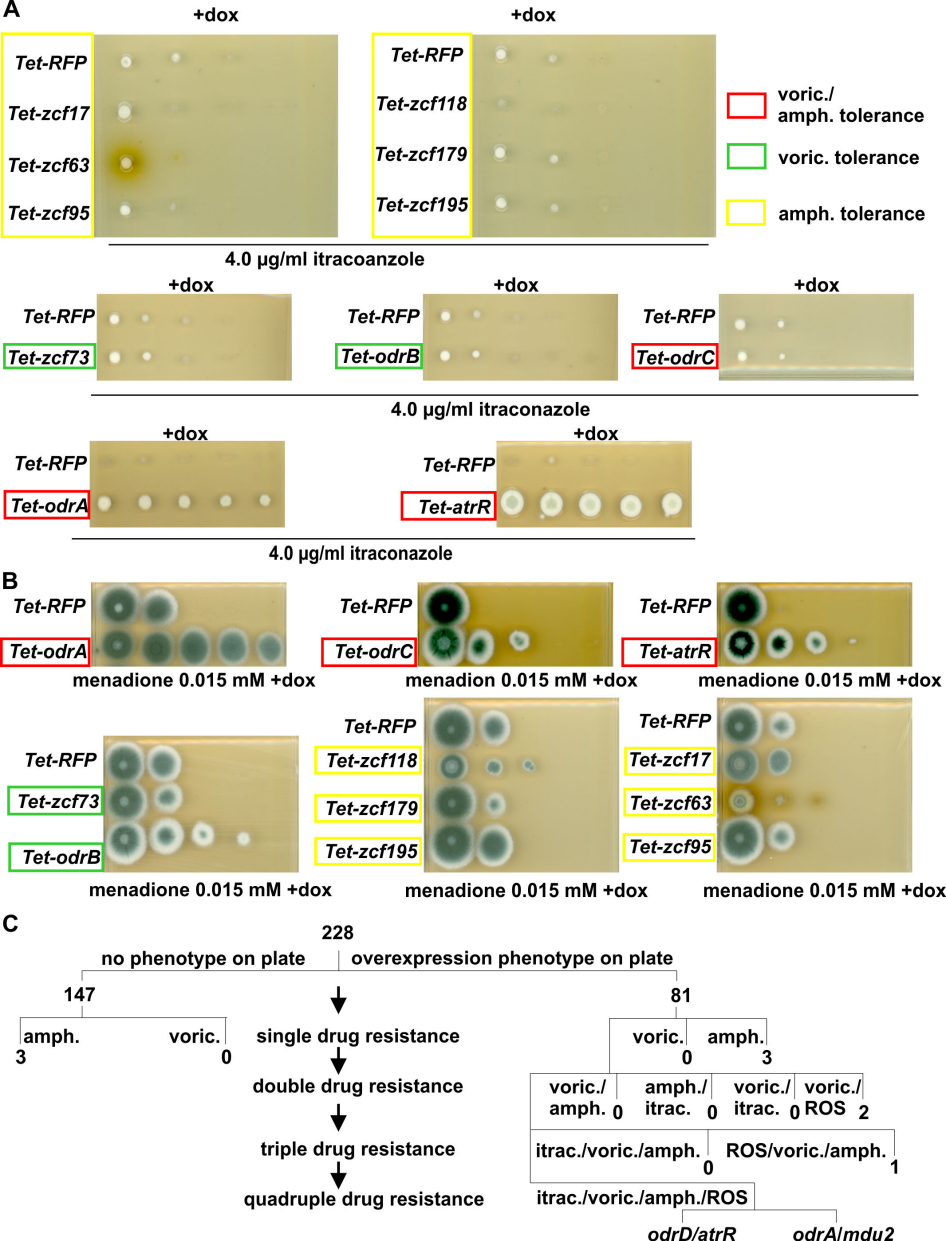
OdrA/Mdu2 and AtrR/OdrD promote increased tolerance to itraconazole, voriconazole, menadione, and amphotericin B. (**A**) Dilution spot tests of the different overexpression strains leading to a resistance to voriconazole, amphotericin B, or both drugs in the presence of itraconazole. Spores were spotted on minimal medium containing 4-µg/mL itraconazole in 1 of 10 dilution steps with a starting amount for the 1.5 × 10^5^ spores. For induction of the *zcf*’s, 50-µg/mL doxycycline was added. The *Tet-RFP* strain was used as control. (**B**) Dilution spot tests of the 11 strains overexpressing *zcf*s, which induce drug resistance in the presence of 0.015 mM menadione. Only the overexpression of *odrA/mdu2*, *odrB*, *odrC*, and *atrR/odrD* led to a highly increased tolerance to menadione. Control and experimental setup was carried out as described for panels A and B. (**C**) Scheme of the screening results of the overexpression library in correlation with drug tolerance. Only two strains, *Tet-odrA* and *Tet- atrR/odrD*, showed a quadruple resistance to amphotericin B, menadione, and the azoles voriconazole and itraconazole.

Drug-resistant clinical isolates of *A. fumigatus* are often cross-resistant to other members of an antifungal group, e.g., against both azoles itraconazole and voriconazole (20, 43). Spot-tests of all 11 overexpression strains in the presence of itraconazole revealed that only overexpression of *odrA*/*mdu2* and *atrR*/*odrD* resulted in strongly decreased susceptibility to both drugs. No combined resistance effects were observed for the other nine strains (Fig. 2A).

Since drug resistance often correlates with oxidative stress response (12, 14, 15, 19, 44), the 11 overexpression strains were tested for menadione resistance (Fig. 2B). Four strains (*Tet-odrA*, *Tet-odrB*, *Tet-odrC*, and *Tet-atrR*) showed oxidative stress and drug resistance (odr). *zcf63* and *zcf118* strains were not included into the *odr* gene group transcription factors since tolerance to menadione was only slightly increased. Within the *odr* genes, only *odrA*/*mdu2* and *odrD* conferred cross-resistance to azoles (quadruple resistance) (Fig. 2C).

In previous works, the transcription factor AtrR/OdrD has been analyzed in more detail, whereas knowledge about the function of OdrA/Mdu2 in *A. fumigatus* is limited (11, 22, 24). Therefore, we focused on the function of OdrA/Mdu2. To confirm the observed resistance to the antifungals voriconazole, itraconazole, and amphotericin B by OdrA/Mdu2, minimal inhibition concentration (MIC) tests were performed in liquid medium (Table S3). Under inducing conditions, the *Tet-odrA* strain showed an increased MIC by one dilution step for all three drugs in comparison to the *Tet-RFP* control strain. Under non-inducing conditions, the MICs were not altered in comparison to the control. This supports our finding of OdrA/Mdu2 as regulator for drug resistance in *A. fumigatus*.

### OdrA/Mdu2 regulates genes involved in stress and detoxification including *mdr1*

Mechanisms for conferring azole resistance of *Aspergillus fumigatus* are often based on increased expression of genes encoding drug efflux pumps or mutations and/or overexpression of *cyp51A* (13, 45). Knowledge on amphotericin B resistance is limited as resistance is multifactorial, including upregulation of genes involved in ROS detoxification or a modified ergosterol pathway (46, 47). We used RNA-seq analysis to investigate how OdrA/Mdu2 influences the response to azoles as well as polyenes. The transcriptomes of the induced *Tet-odrA* and the *Tet-RFP* control overexpression strains were compared. RNA-seq data were validated by qPCR experiments (Fig. S8A). Genes showing a log2 fold change of ≥1 in expression and a *P* value of < 0.05 were considered as differentially expressed genes (DEGs).

**Fig 3:**
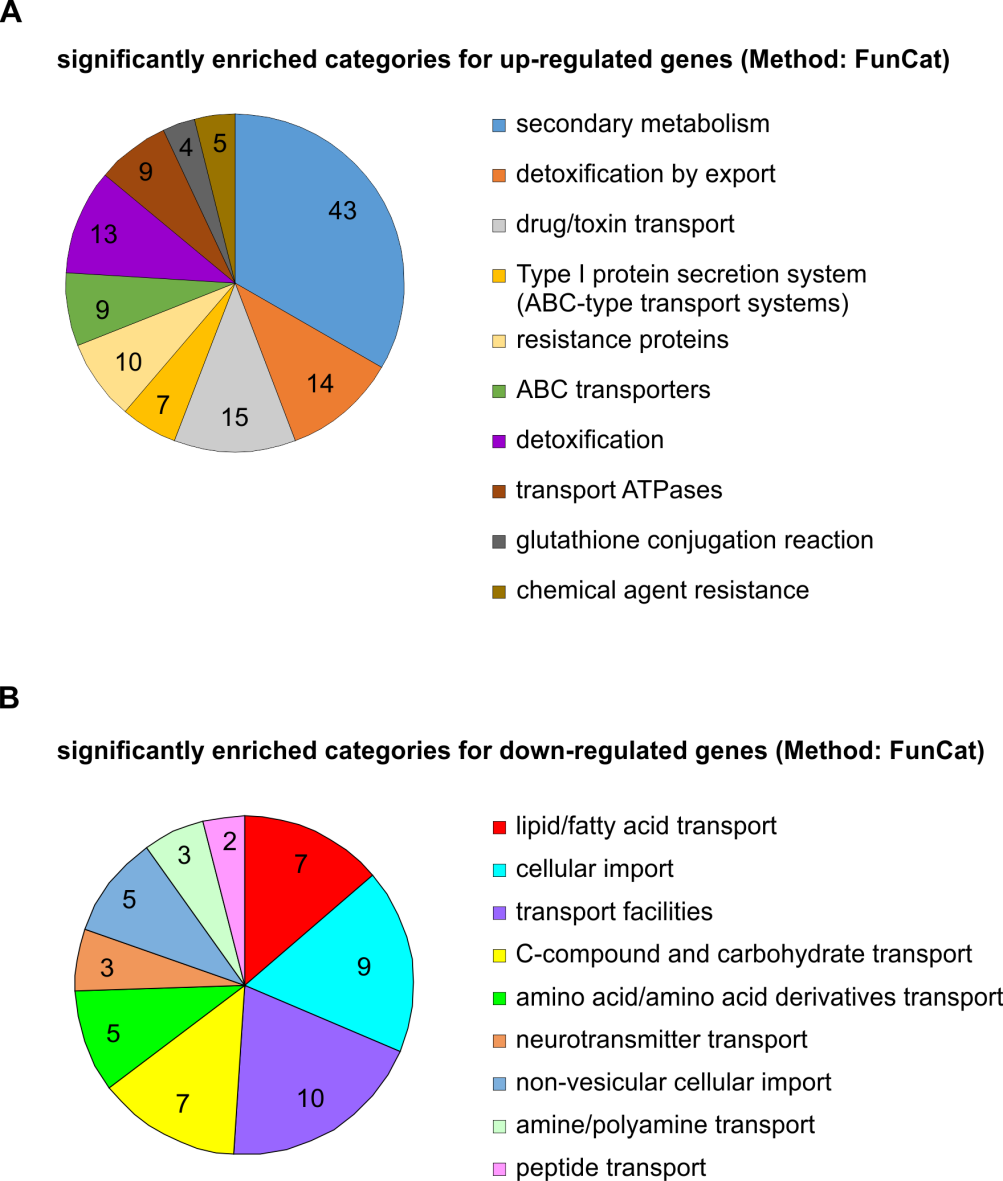
FunCat classification of OdrA/Mdu2 regulated genes. RNA-seq analysis of the *Tet-odrA* overexpression strain reveals increased expression of genes involved in detoxification and stress response. RNA-seq analysis of the *Tet-odrA* strain in comparison to the *Tet-RFP* control strain. Strains were incubated in liquid minimal medium for 18 hours and afterward shifted to fresh medium containing 50-µg/mL doxycycline for induction. Three biological replicates were used for sequencing. (**A and B**) Categorization of the different regulated genes in dependency of OdrA/Mdu2. Genes were classified in functional groups based on the FunCat database. Groups of upregulated genes with a value of ≥2 fold change (FC) are shown in panel **A**. (**B**) Functional groups of the downregulated genes with a value of ≥2 FC.

A total of 280 identified DEGs included 218 upregulated and 62 downregulated genes (Fig. 3; Table S4A through E). This suggests that OdrA/Mdu2 acts primarily as a transcriptional activator. Functional categorization (FunCat) analysis of DEGs revealed that upregulated genes are enriched for groups connected with detoxification and stress response (*P* value of <0.05). However, none of these genes is known to be directly involved in ergosterol biosynthesis. Among the 62 downregulated genes, 9 different FunCat groups were enriched and correlated with cellular import and uptake systems (*P* value of <0.05). These data imply that multi-drug resistance caused by OdrA/Mdu2 is based on a combination including activation of genes required for detoxification and the simultaneous inhibition of genes encoding uptake systems to counteract the accumulation of toxic compounds.

Azole tolerance can be caused by mutations of *cyp51A* or by increased expression of drug transporters (13). Upon overexpression of *odrA/mdu2*, five genes encoding the transporters AbcD, AtrI, Abc1, Abc3, and Mdr1 showed significantly increased expression levels (Fig. 3A; Fig. S8B; Table S4A) and were previously connected to azole tolerance (11, 21, 48). Overexpression strains for these five transporter genes were constructed using the *TetOn* system to explore whether OdrA/Mdu2-induced azole resistance is mediated by upregulation of these genes. Increased *abc1*, *atrI*, or *abcD* transcript levels did not affect the tolerance to voriconazole or itraconazole, whereas elevated *abc3* transcripts resulted in a slightly increased tolerance to voriconazole (Fig. S7). However, this effect is much smaller than the observed tolerance to OdrA/Mdu2 overexpression. Only increased *mdr1* expression provided selectively increased itraconazole tolerance similar to the *odrA*/*mdu2* overexpression strain (Fig. S7C). However, increased *mdr1* expression did not provide simultaneous voriconazole tolerance, which therefore depends on a different molecular mechanism. Consistently, the deletion of *mdr1* in the *Tet-odrA* strain background diminished the tolerance to itraconazole but not to voriconazole (Fig. S7D). These observations support earlier findings that *mdr1* is required for itraconazole but not for voriconazole tolerance (20).

### OdrA*/*Mdu2 regulates the *mdr1* and *atrR*/*odrD* expression directly

To test if OdrA/Mdu2 could bind in the upstream region (3 kb) in proximity to *mdr1* and thus be able to directly regulate its expression, a chromatin immunoprecipitation (ChIP) experiment followed by ChIP qPCR was carried out. We used a *Tet-odrA-GFP* strain producing a high amount of a functional OdrA-GFP fusion protein (Fig. S8A and B) alongside a *GFP* overexpressing strain, which served as a negative control. Three different primer pairs were used for promoter analysis by qPCR. The respective regions were chosen based on CGG repeats, which are common recognition sites for this group of regulators (26). A significant enrichment of region 2 (22.0-fold enrichment) and region 3 (14.6-fold enrichment) was observed in comparison to the control. In contrast, no enrichment of OdrA-GFP compared to free GFP was observed for the more distant region 1 (Fig. S8C and D). Our data strongly suggest that OdrA/Mdu2 regulates the expression of *mdr1* by direct binding to its promoter.

**Fig 4:**
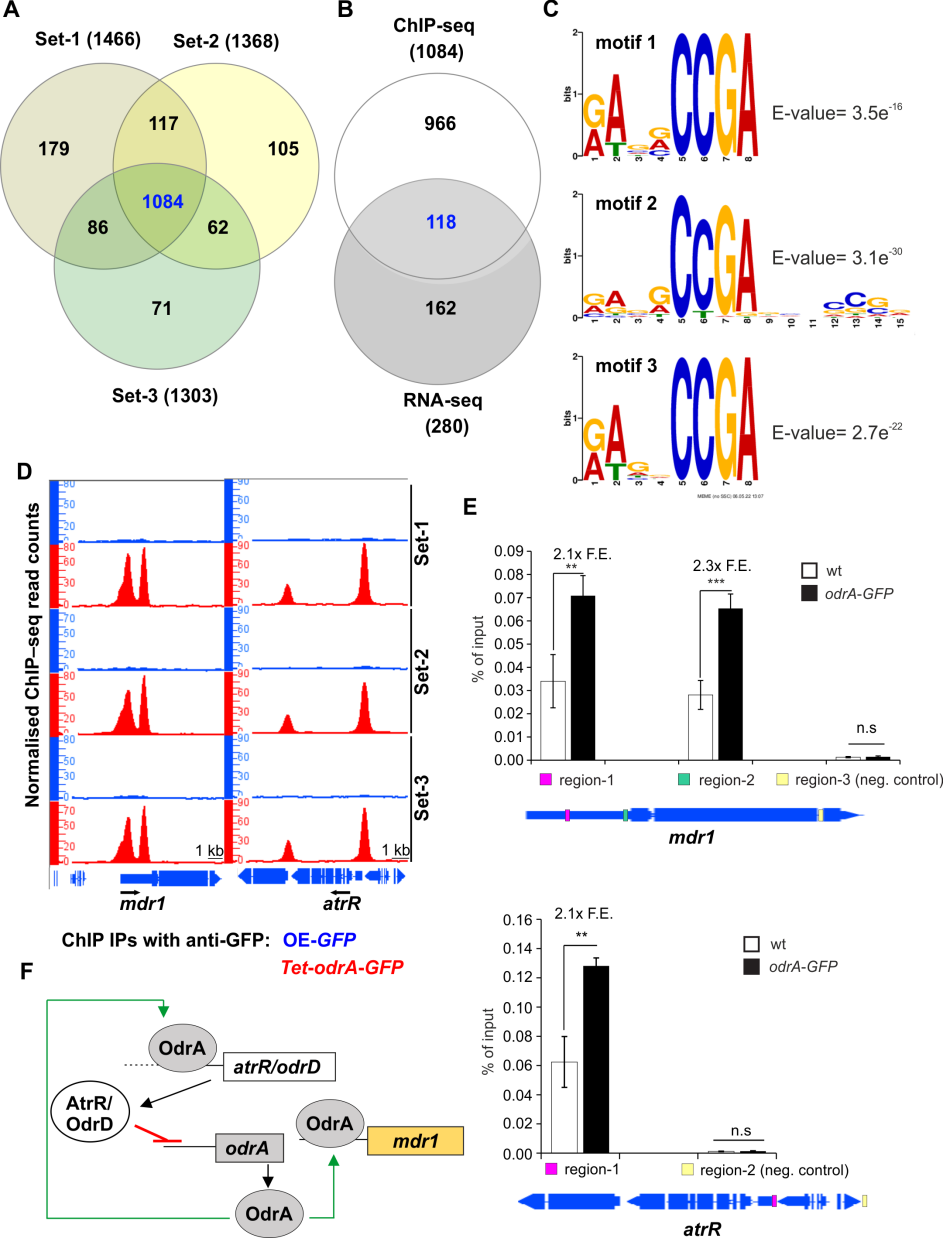
ChIP-seq analysis reveals the direct target genes of OdrA-GFP in *A. fumigatus*. (**A**) Venn diagram presenting the overlap of three independent sets of ChIP-seq analysis (OE-*GFP* versus *Tet-odrA-GFP*). A total of 1,084 locus IDs of genes associated with ChIP-seq peaks were found in these sets. Each set derives from the analysis of different biological replicates of the corresponding samples. (**B**) Venn diagram presenting 118 genes in the intersection between the 1,084 ChIP-seq peaks (as described in panel A) with the DEGs of the RNA-seq (with cutoffs of log2(FC) <−1 and >1). (**C**) *De novo* motif discovery by the MEME-ChIP tool. The input was 100-bp sequences spanning below the summit of the top 150 ChIP-seq peaks, found within the 3-kb promoter regions, for each independent set of the ChIP-seq analysis. (**D**) Screenshots from the Integrated Genome Browser, representing the direct *in vivo* binding of OdrA*-*GFP upstream from the open reading frames (ORFs) of selected genes; black arrows depict the direction of the transcription. (**E**) ChIP qPCR experiments for binding studies of OdrA/Mdu2 at promoter regions of *mdr1* and *atrR/odrD* under native conditions. We used the wild type as control. The diagram shows the regions used for PCR and the fold enrichment (F.E.) in comparison to the control. We used regions outside the promoter regions as internal negative controls. For both genes, we observed a binding of OdrA/Mdu2. For *mdr1* ChIP qPCRs, we used the primers MB1644 and 1645 for region 1, MB1646 and MB1647 for region 2, and MB1648 and MB1649 for region 3 (negative control). For *atrR/odrD*, we used the primers MB1650 and MB1651 for region 1, and MB1652 and MB1653 for region 2 (negative control). (**F**) Scheme of the regulatory network of OdrA/Mdu2 by binding at the *atrR*/*odrD* promoter. Red lines indicate a repressive effect, whereas green arrows mark increasing expression. **, *P* < 0.01; ***, *P* < 0.001; n.s., not significant; wt, wild type.

Previous work (24) identified AtrR/OdrD as repressor of *odrA*/*mdu2* and *mdr1* expression. RNA-seq data revealed that *mdr1* and *atrR*/*odrD* are upregulated by *odrA*/*mdu2* overexpression (Fig. 3; Table S4A). OdrA/Mdu2 binds to the *mdr1* promoter, suggesting a direct regulation (Fig. S8). To identify the complete set of OdrA/Mdu2 target genes, a ChIP-seq experiment using the *Tet-odrA-GFP* and the *GFP* overexpression strains was performed. Analysis of ChIP-seq data revealed that OdrA/Mdu2 binds to 1,084 regions within the genome of *A. fumigatus* (Fig. 4A and B; Table S5A through D). Comparison of these regions with the RNA-seq data results in an overlap of 118 genes (Fig. 4B), including *atrR*/*odrD* and *mdr1* (Fig. 4C). Out of the overlapping peaks from the ChIP-seq data sets, the 150 top scoring peaks, found within the 3-kb promoter regions, were analyzed using MEME for *de novo* motif discovery. Three similar promoter binding motifs for OdrA/Mdu2 were identified, comprising eight nucleotides, where the last four are highly conserved (CCGA) (Fig. 4D). ChIP-seq data derived from overexpression strains might include false positive binding regions. For further validation, ChIP qPCR experiments were carried out using a strain expressing *odrA-GFP* under its native promoter. We used the wild-type strain as negative control. Binding of OdrA/Mdu2 to the promoter regions of *atrR*/*odrD* and *mdr1* was analyzed based on the ChIP-seq data. For both genes, it was shown that OdrA-GFP directly binds to the regions that were previously discovered (Fig. 4D and E). In contrast, PCRs with primers annealing outside of the *mdr1* and *atrR*/*odrD* promoters did not show a significant enrichment. Our data indicate that OdrA/Mdu2 directly regulates *mdr1* and *atrR*/*odrD*.

Comparison of *atrR*/*odrD* (24) and *odrA*/*mdu2* RNA-seq data sets revealed that, beside *atrR*/*odrD*, 11 out of the 13 genes displaying an altered expression in the *atrR*/*odrD* deletion strain are direct targets of OdrA/Mdu2 (Table 1). This suggests that AtrR/OdrD regulates a subset of genes like *mdr1* by controlling the expression of *odrA*/*mdu2*.

**TABLE 1:** Subset of genes regulated by AtrR/OdrD and OdrA/Mdu2

Gene		Regulation		Binding peaks of OdrA/Mdu2 upstream (−) or downstream (+) of the ATG start codon
Number	Name	Δ*atrR*	*Tet-odrA*	
Afu1g03800	*odrA/mdu2*	Up	Up	not found
Afu2g05060	*aoxA*	Up	Up	842
Afu3g13010		Up	Up	453
Afu4g09450		Up	Up	246
Afu4g09450		Up	Up	+753 (inside the 2nd intron)
Afu4g14380		Up	Up	247
Afu5g06070	*mdr1*	Up	Up	1668
Afu7g00950		Up	Up	1038
Afu8g01860		Up	Up	181
Afu8g06430		Up	Down	Not found
Afu2g02690	*atrR/odrD*	−	Up	811
Afu3g01910		Down	Up	152
Afu3g01910		Down	Up	+1447 (inside the seventh exon)
Afu5g14740	*fleA*	Down	Down	Not found
Afu6g03450		Down	Up	340

In summary, our data suggest that AtrR/OdrD-dependent repression of *mdr1* transcription is indirect and mediated by repressing *odrA*/*mdu2* expression. Induction of OdrA/Mdu2 increases the expression of *mdr1* by direct binding. At the same time, OdrA/Mdu2 induces the expression of *atrR*/*odrD* by binding at its promoter region, leading to connected regulatory networks between AtrR/OdrD and OdrA/Mdu2 (Fig. 4F).

### Basal expression levels of *odrA*/*mdu2* are required for natural resistance to amphotericin B, voriconazole, and menadione but not to itraconazole

**Fig 5:**
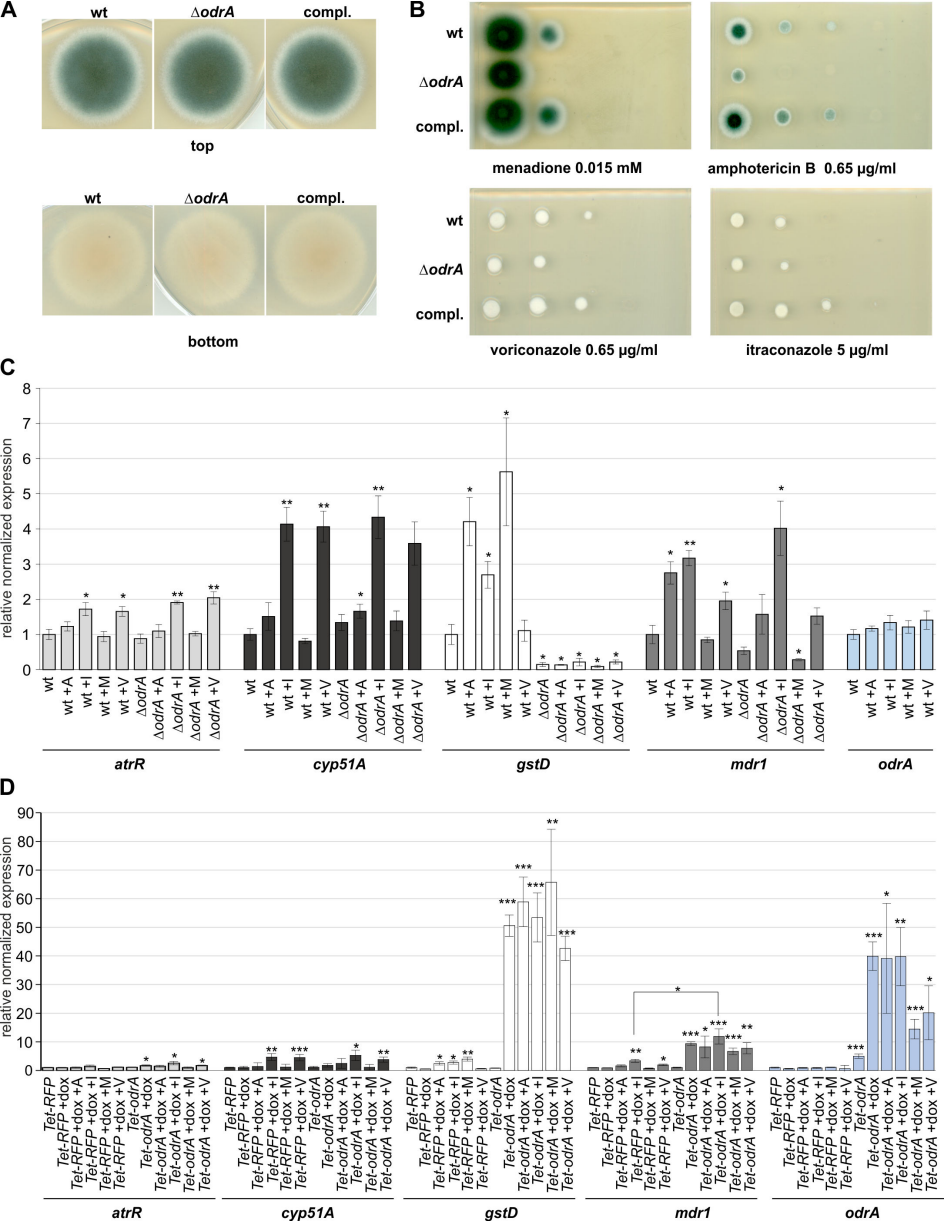
Influence on the transcriptional level of *odrA/mdu2* and its target genes by antifungal drugs and oxidative stress. (**A**) Spot tests of the wild-type (wt), the *odrA* deletion strain (∆*odrA*), and the complementation (compl.). Approximately 2,000 spores were spotted on minimal medium. Plates were incubated for 3 days at 37°C. Absence of *odrA/mdu2* does not affect growth, color, or conidiospore formation in *A. fumigatus* in comparison to the wild type. (**B**) Dilution spot test of the wt, the ∆*odrA*, and the compl. strain on minimal medium containing amphotericin B, voriconazole, itraconazole, or menadione. Minimal medium without any drug was used as control. Spores were spotted in 1 of 10 dilution steps. The starting amount for the spot tests was 1.5 × 10^5^ spores. The absence of the *odrA/mdu2* gene increases the susceptibility to amphotericin B, voriconazole, and menadione. For itraconazole, no differences between wild-type and deletion strain could be observed. All effects could be complemented by reintegration of the *odrA/mdu2* gene in the deletion strain. (**C and D**) qPCR experiments of the wt and the ∆*odrA* (**C**) or the *Tet-RFP* and the *Tet-odrA* overexpression strain (**D**). Strains were incubated with and without drugs (**C and D**). (**D**) Strains were incubated for 18 hours in liquid minimal medium at 37°C. Afterward, amphotericin B (abbreviated with “A,” final concentration [conc.] 0.65 µg/mL), itraconazole (abbreviated with “I,” final conc. 5 µg/mL), voriconazole (abbreviated with “V,” final conc. 0.65 µg/mL), or menadione (abbreviated with “M,” final conc. 0.04 mM) was added for an additional 4 hours. For induction of the *Tet* system, 50-µg/mL doxycycline was added for 4 hours as well. Four biological replicates were used. For normalization, the housekeeping genes *H2A* and *eIF2B* were used. Significance was calculated using Student`s *t*-test. *, P < 0.025; **, *P* < 0.0025; ***, *P* < 0.00025. Error bars represent the standard error of the mean.

Overexpression of *odrA*/*mdu2* increases the tolerance to voriconazole, itraconazole, and to amphotericin B (Fig. 1 and 2C). An *odrA*/*mdu2* deletion strain was generated to examine whether loss of *odrA*/*mdu2* correlates with increased susceptibility to these antifungal drugs. The ∆*odrA* strain displayed no obvious phenotypical alterations in comparison to the wild-type or complementation strain in the absence of drugs but leads to an increased susceptibility in the presence of voriconazole, menadione, or amphotericin B (Fig. 5A). In contrast, the susceptibility to itraconazole was not affected. Reintegration of *odrA*/*mdu2* into the genome of the ∆*odrA* strain suppressed these effects (Fig. 5B). To further investigate the regulatory network of OdrA/Mdu2, qPCR experiments were carried out using the wild-type and the *odrA*/*mdu2* deletion strains in the absence and the presence of drugs (Fig. 5C). The expressions of *odrA*/*mdu2* itself and of four target genes, which are directly affected in ChIP-seq experiments by OdrA/Mdu2, were analyzed. *odrA*/*mdu2* expression itself is not influenced in the presence of drugs. Transcripts of *mdr1*, *AFUA_4* G14,380 (encoding a putative glutathione-S-transferase, named as *gstD*), and *atrR*/*odrD* showed increased expression in the RNA-seq data set. *cyp51A* was found in ChIP-seq experiments but was not regulated by OdrA/Mdu2 (Table S5D). No differences in the expression level of *atrR*/*odrD* were observed between wild-type and *odrA/mdu2* deletion strains under all tested conditions. *cyp51A* levels were moderately increased in the deletion strain compared to the wild-type strain in the presence of amphotericin B (Fig. 5C). Absence of *odrA*/*mdu2* results in less than twofold increased induction of *cyp51A* compared to wild type and thus seems not to be responsible for the observed resistance, although reduced ergosterol levels can result in decreased susceptibility to amphotericin B (49, 50). In contrast, *mdr1* is regulated in an OdrA/Mdu2-dependent manner in the presence of amphotericin B or menadione but not with voriconazole or itraconazole. The OdrA/Mdu2 impact on *gstD* expression is even stronger under all tested conditions except in presence of voriconazole. Absence of *odrA*/*mdu2* leads to decreased *gstD* expression (Fig. 5C).

Overexpression of *odrA*/*mdu2* significantly increases the expression of *atr*R/*odrD*, *gstD*, and *mdr1* without drugs and partially in the presence of drugs in comparison to the control. In contrast, overexpression of *odrA*/*mdu2* does not affect the *cyp51A* expression level (Fig. 5D). Increased *cyp51A* expression in the presence of itraconazole and voriconazole is independent of *odrA*/*mdu2* because this effect is observed in the *Tet-RFP* control and the *odrA*/*mdu2* deletion strain, respectively (Fig. 5C and D).

Our data suggest that OdrA/Mdu2 regulates different subsets of genes, depending on the conditions. Some genes like *atrR*/*odrD* are only regulated when *odrA*/*mdu2* is overexpressed, whereas other genes are already controlled in the presence of certain drugs (*mdr1*) or even under native conditions without drug treatment (*gstD*).

### Menadione and amphotericin B increase nuclear OdrA/Mdu2 accumulation for activation of stress and drug response genes

**Fig 6:**
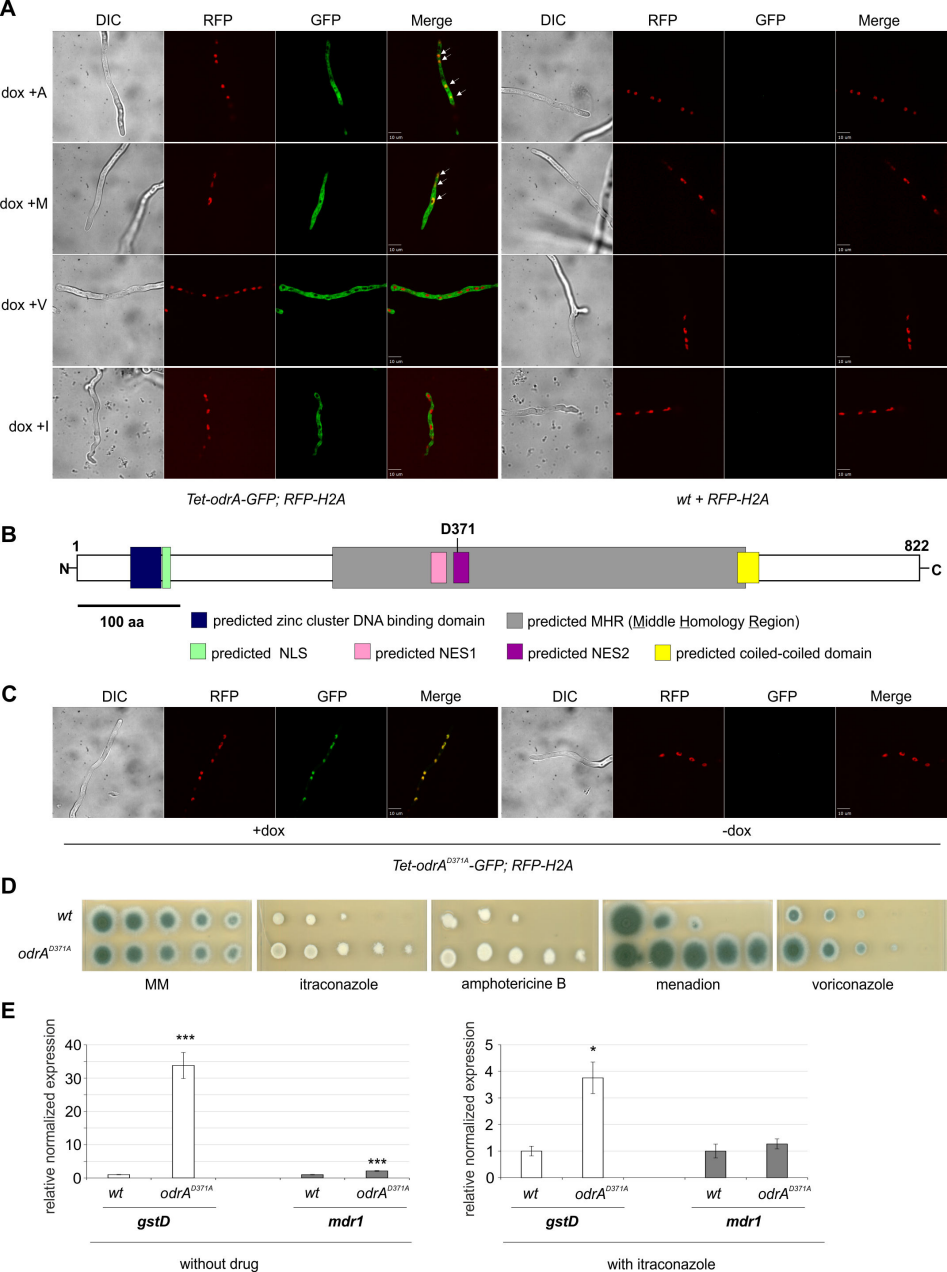
Oxidative stress and amphotericin B lead to the accumulation of OdrA/Mdu2 in the nucleus. (**A**) Microscopy of the *Tet-zcf46-GFP* strain and the wild type. Strains were grown in minimal medium containing 5-µg/mL doxycycline overnight. Afterward, strains were incubated for an additional 4 hours in the presence of amphotericin B (abbreviated with “A,” final conc. 0.65 µg/mL), itraconazole (abbreviated with “I,” final conc. 5 µg/mL), voriconazole (abbreviated with “V,” final conc. 0.65 µg/mL), or menadione (abbreviated with “M,” final conc. 0.04 mM). Nuclei were visualized by an RFP tagged H2A protein in the corresponding strains (red fluorescence). Yellow fluorescence indicates a co-localization of H2A and OdrA/Mdu2 indicated a nuclear localization of the transcription factor (indicted by white arrowheads). (**B**) Scheme of the OdrA/Mdu2 protein with the predicted domains based on FungiDB (42). (**C**) Microscopy of the *Tet-zcf46^D371A^-GFP* strain, which has an amino acid exchange in its NES. The strains were grown overnight with or without 5-µg/mL doxycycline. Nuclei were visualized by an RFP tagged H2A protein in the corresponding strains. (**D**) Dilution spot tests of the *odrA*^D371A^ and the wild-type strain on minimal medium and minimal medium with itraconazole, amphotericin B, voriconazole or menadione. Spores were spotted in 1 of 10 dilution steps with a starting amount for the 1.5 × 10^5^ spores. (**E**) qPCR experiments of the wild type (wt) and the *odrA*^D371A^. Strains were incubated in the presence or absence of 5-µg/mL itraconazole. Strains were grown for 18 hours in liquid minimal medium at 37°C and afterward incubated in the presence or absence of 5-µg/mL itraconazole for an additional 4 hours. Three biological replicates were used. For normalization, the housekeeping genes *H2A* and *eIF2B* were used. Significance was calculated using Student`s *t*-test. *, *P* < 0.025; ***, *P* < 0.00025. Error bars represent the standard error of the mean.

Post-transcriptional mechanisms might influence OdrA/Mdu2-mediated antifungal drug responses because *odrA*/*mdu2* expression is unchanged during the tested stress conditions (Fig. 5C). Microscopic localization studies were carried out to explore whether OdrA/Mdu2 transcription factor activity is based on shuttling of the regulator between nucleus and cytoplasm (27, 51–53). A *Tet-odrA-GFP* strain was induced with low concentrations of doxycycline (5 µg/mL) because OdrA/Mdu2 is not visible when expressed under its native promoter. Wild type with RFP-labeled H2A served as control. No increased accumulation in the nucleus was observed (indicated by the red fluorescence of the nuclei and green fluorescence in the hyphae) in the presence of doxycycline alone or in combination with one of the two azoles, itraconazole or voriconazole. In contrast, amphotericin B and menadione induced the accumulation of OdrA/Mdu2 in the nucleus (indicated by the yellow color of the merged GFP and RFP fluorescence) (Fig. 6A; Fig. S9A). These results show that nuclear import/export balance of OdrA/Mdu2 is altered in dependency of stress. Analysis of the protein sequence for predicted nuclear export signal (NES) and nuclear localization signal (NLS) using the NLStradamus and the LocNES tool (54, 55) identified one putative NLS and two putative NESs (NES1: aa 349–363, score of 0.383; NES2: aa 371–385, score of 0.400) (Fig. 6B). Amino acid exchange at position 371 from aspartic acid (D) to alanine (A) in NES2 led to a strong accumulation of OdrA/Mdu2 in the nucleus in the absence of any drug (Fig. 6C). This indicates that NES2 is functional and required for nuclear OdrA/Mdu2 export. Insertion of this mutation into the wild-type strain leads to increased resistance to all tested substances compared to the non-mutated version (Fig. 6C). Further, the combination of overexpression and NES2 mutation did not increase the resistance in comparison to overexpression alone (Fig. S9B). Nuclear accumulation finally resulted in *gstD* and other target gene activations. However, *mdr1* expression was only significantly increased in the absence but not in the presence of itraconazole. This indicates that nuclear accumulation without overexpression of *odrA*/*mdu2* induces itraconazole resistance independently of *mdr1*. In contrast, overexpression of OdrA/Mdu2 leads to a significantly increased expression of *mdr1* compared to the control strain, even in the presence of itraconazole (Fig. 5D). This strongly suggests that itraconazole resistance caused by OdrA/Mdu2 is related to *mdr1* regulation but also involves additional factors.

In summary, we could show that OdrA/Mdu2 is a stress response regulator which reacts to oxidative stress signals like amphotericin B and menadione by nuclear accumulation, resulting in the activation of genes involved in adaption to environmental stress.

## DISCUSSION

The potential for rewiring transcriptional genetic networks is an important prerequisite for fungal survival as a response to drastic environmental changes. This includes the reaction not only to cell-damaging amounts of antimycotics but also to other stress conditions. Aspergilli produce stress-related nuclear transcription factors already during vegetative growth in anticipation of upcoming challenging conditions. In parallel, they also produce the corresponding protein destruction machinery in case these transcription factors are not needed (56, 57). Antifungals like polyenes produced by other microbes challenge the opportunistic pathogen *A. fumigatus* during saprophytic growth in natural environments such as soil and during medical treatment of infected patients. Environmental drivers might play an important role in acquiring and evolving fungal drug resistance (58). Special modifications or artificial activation can result in increased drug tolerance as shown for Mrr1 in *C. albicans* (59). Another commonly used strategy for mounting of stress responses in microorganisms is the increased expression of transcriptional regulators. Therefore, the generated overexpression library comprising 228 *zcf* genes of *A. fumigatus* represents a useful tool for the analysis of zinc cluster transcription factors in correlation with stress and drug responses and complements the existing *A. fumigatus* deletion libraries (32, 60, 61).

Overexpression of 11 of the 228 *zcf* genes encoding fungal zinc cluster transcription factors promoted increased tolerance to polyenes and azoles. These genes are distinct from more than 400 deleted genes encoding transcription factors, which provide itraconazole susceptibility to corresponding mutant strains (31). Only *atrR*/*odrD* was identified in both screens, providing further validity of our approach. A combined tolerance against amphotericin B and voriconazole was observed for three genes (*odrA*/*mdu2*, *odrC*, and *atrR*/*odrD*).

OdrA/Mdu2 and AtrR/OdrD are special because overexpression influences the tolerance not only to voriconazole, amphotericin B, and to the oxidative stress generating compound menadione but also to itraconazole. Such a specific combined tolerance against amphotericin B with itraconazole has not been described before. OdrA/Mdu2 and AtrR/OdrD are part of the *odr* subgroup of *zcf* genes (*odrA-D*) and control each other in a mutual regulation where OdrA/Mdu2 activates the AtrR/OdrD encoding gene, whereas vice versa, AtrR/OdrD functions as gene repressor. The details of the molecular mechanism of this interplay between AtrR/OdrD and OdrA/Mdu2 will be an interesting task for the future as well as the comparison with similar transcription factors in other fungi including *A. nidulans* (e.g., AflR and ClcA), *C. albicans*, or *Neurospora crassa* (26, 28, 62, 63).

**Fig 7:**
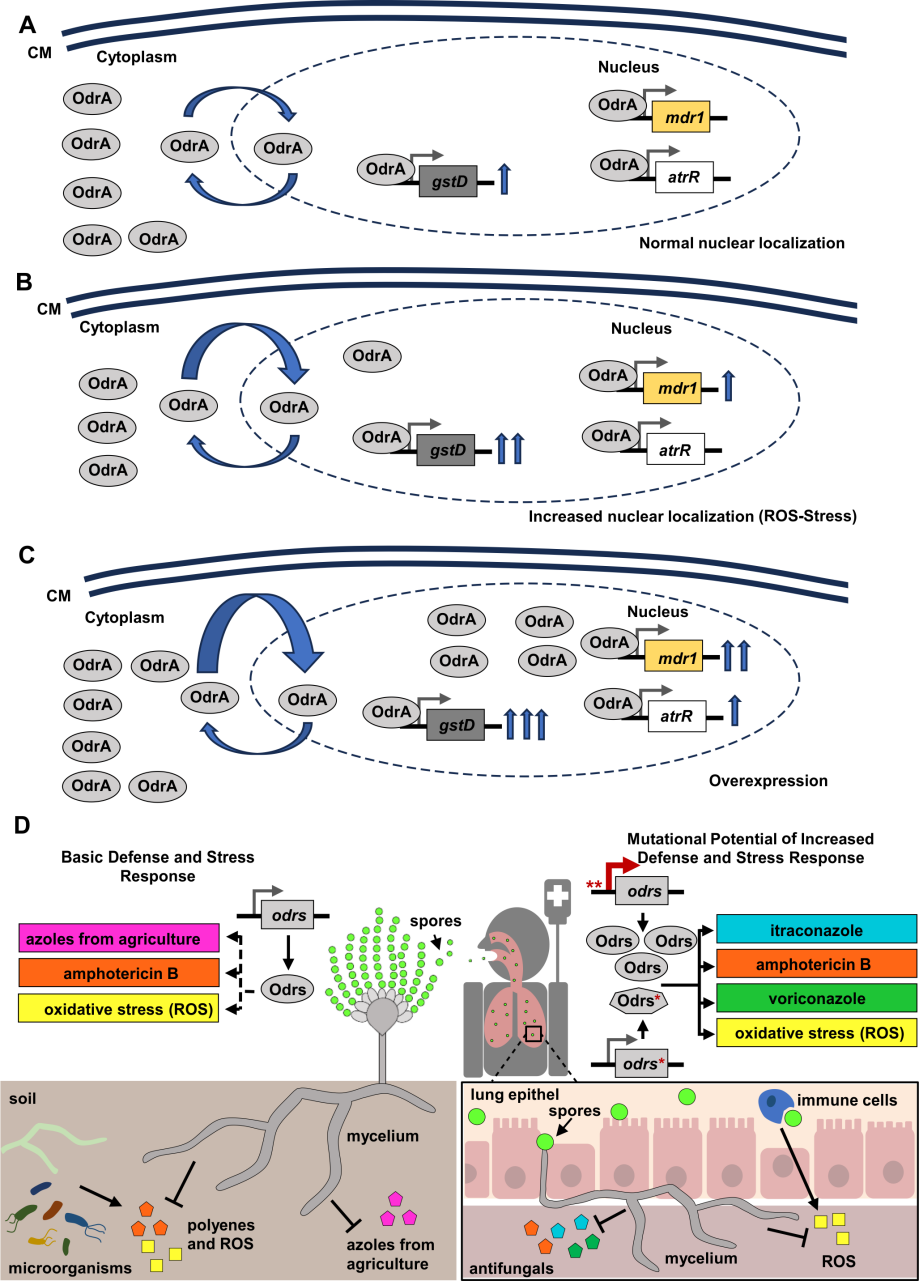
OdrA/Mdu2 as part of a multidrug response system which is induced by oxidative stress. (**A–C**) Model of the regulation and influence of OdrA/Mdu2 on stress and drug resistance. In this process, OdrA/Mdu2 regulates different genes with varying intensity, depending on the external conditions. The number of arrows indicates the expression level. The basal expression is sufficient for natural adaptation to azoles such as voriconzole (**A**). In the presence of oxidative stress, either directly through ROS or indirectly through polyenes such as amphotericin B, the accumulation of OdrA/Mdu2 in the nucleus activates further genes (**B**). Overexpression then causes already activated genes to be transcribed even more strongly, and new genes such as *atrR/odrD* are activated (**C**). To simplify the figure, only AtrR/OdrD and OdrA/Mdu2 were used instead of AtrR/OdrD and OdrA/Mdu2. (**D**) Odr’s are required as basic defense and stress response of *A. fumigatus* within the soil. They are part of defense systems, which enable the adaption to toxic compounds released from microorganisms and protects against azoles commonly used in agriculture (left panel). Gain of function mutations and/or mutations in the promoter, indicated by asterisks, lead to a higher or stabilized amount of Odrs, resulting in an increased stress response, which can even increase the resistance spectrum to antifungals. The induced stress response system enables the fungus to better adapt to harsh conditions, which could be found in the human host (right panel). Dashed arrows indicate a normal wild-type response, whereas solid arrows indicate an increased stress response. Spores are shown in green. The color code used for the different drugs in the upper part of the figure correlates with colors used in the lower part.

*A. fumgigatus* OdrA/Mdu2 regulates a large set of stress response genes like *gstD* and *mdr1* (Fig. 3). Thereby, the resistance caused by OdrA/Mdu2 seems rather multifactorial than limited to few specific genes. In addition to the induced resistance by overexpression of OdrA/Mdu2, also the nuclear enrichment of this regulator reduces drug susceptibility (Fig. 7). This effect was visible in the presence of amphotericin B and menadione, which both lead to oxidative stress in the cell (64–66). This suggests that the increased nuclear localization induced by these substances occurs via the same mechanism. Thus, the observed amphotericin B resistance is based on increased oxidative stress adaptation rather than a specific response to the polyene itself. Previous work showed that azoles like itraconazole induce oxidative stress as well (67). A pronounced accumulation of OdrA in the nucleus in the presence of the tested azoles was not observed. One explanation is that the oxidative stress induced by this group of drugs is not strong enough to foster nuclear accumulation of OdrA/Mdu2 or that the shift of this regulator only occurs through specific ROS signals.

In contrast to OdrA/Mdu2 and AtrR/OdrD, overexpression of *zcf95*, *zcf179*, and *zcf195* confers high resistance to amphotericin B but not to menadione, indicating a ROS independent drug response system. Presumably, this relates to a different response pathway that does not directly lead to growth deficits under asexual conditions. This resistance might be connected with modifications of the cell wall as described for *Candida* species (68).

The OdrA/Mdu2-dependent regulation of its target genes correlates with the expression level of *odrA*/*mdu2* and its nuclear accumulation induced in the presence of ROS and antifungal drugs (Fig. 7A and B). Thus, some genes such as *gstD* require OdrA/Mdu2 already for basal expression (Fig. 7A). Other genes like *mdr1* are only regulated under stress conditions, when OdrA/Mdu2 accumulates in the nucleus or upon high expression levels of the transcription factor itself, leading to the regulation of *atrR*/*odrD* (Fig. 7B and C). Thus, conditional regulation of OdrA/Mdu2 target genes enables *A. fumigatus* to specifically tailor the genetic program to changing conditions.

The identified OdrA-D encoding gene group is part of a potent and powerful defense system which coordinates multiple levels of protection mechanisms against bacterial competitors, the host immune system, and antifungal drug treatment during aspergillosis (Fig. 7D). Consequently, gain of function or promoter mutations of the Odr regulators presumably potentiate the pathogenicity of *A. fumigatus* similar to OdrA/Mdu2. Corresponding mutations may arise within the host or in the natural environment under harsh conditions. It will be an important future task to increase our understanding how these Odr systems are controlled to identify potential Achilles heels. This study serves as an initial step to develop according counter measures against strains with increased resilience against antifungal drug treatment and immune responses inside the human host.

## MATERIALS AND METHODS

### Strains, media, and growth conditions

*Escherichia coli* DH5α strain was used for general cloning purposes (69) and was cultivated in lysogeny broth (LB) medium (1% Bacto-tryptone, 0.5% yeast extract, 1% NaCl, pH 7.5) supplemented with either ampicillin (100-µg/mL final concentration [conc.]) or kanamycin (50-µg/mL final conc.). For cultivation of *A. fumigatus*, minimal medium (MM) (1% glucose, 10 mM NaNO_3_, 20-mL/L salt solution [348 mM KCl, 105 mM MgSO_4_, 558 mM KH_2_PO_4_, 50-mL/L trace elements {70}], adjusted with NaOH at pH 6.5) was used, if not stated otherwise. For solid medium, 2% agar was added. Pyrithiamine was supplemented in a final concentration of 150 ng/mL for selection. Used and constructed strains are listed in Table S6. Used and constructed plasmids are listed in Table S7, and used primers are listed in Table S8.

### Transformation procedures

*E. coli* transformation was carried out according to Hanahan et al. (71). *A. fumigatus* transformation followed the protocol by Käfer (72).

### Manipulation of nucleic acid and purification

For PCR amplification Phusion polymerase (Thermo Fisher Scientific, Waltham, USA) was used. Sanger sequencing was carried out at Seqlab Göttingen/Microsynth AG. Plasmid DNA was isolated using the NucleoSpin Plasmid kit (Macherey-Nagel, Düren, Germany). For gel extraction, the NucleoSpin Gel and PCR Clean-up Kit (Macherey-Nagel) was used. Isolation of genomic DNA of *A. fumigatus* was carried out as described by Kolar et al. (73). The Geneart Seamless Cloning and Assembly kit (Thermo Fisher Scientific) was used for plasmid construction and cloning steps, or by standard cloning protocols using a T4 ligase (Thermo Fisher Scientific). All kits and enzymes were used according to the user’s manual.

### Southern hybridization

Southern hybridization was carried out as described (74). For probe labeling, the AlkPhos Direct Labeling Module (GE Healthcare Life Technologies, Little Chalfont, UK) was used according to the user’s manual.

### BLAST search

The protein sequence of the zinc cluster DNA binding domains (accession no. cd00067, NCBI [40]; accession no. IPR036864, CADRE/EnsemblFungi [41]) was used for a blastp search in the *A. fumigatus* strain Af293 (75). A total of 228 proteins were identified containing an annotated zinc cluster domain. All 228 candidates were used for the construction of the overexpression library.

### Plasmid construction for the overexpression library

The *TetOn* overexpression plasmid pChS3 was constructed in two steps: a fragment including the 5′UTR of *pyroA* and the coding sequence of *pyroA* was amplified using the oligonucleotides pyroA-1 and pyroA-2. The restriction sites *Hpa*I, *Psi*I, *Nhe*I, and *Ssp*I were integrated by pyroA-1. Genomic DNA of strain Af293 was used as template. The PCR-amplified 2.6-kb fragment was integrated via *Eco*RV in the pBluescript II SK (+) to receive the vector pChS02. In the second step, the 3′UTR of *pyroA* was PCR-amplified using the primers pyroA-3 and pyroA-4 to receive a 1.6-kb fragment. The pyroA-4 primer contains the sequence for the restriction sites *Hpa*I, *Psi*I, *Nhe*I, and *Ssp*I. Furthermore, the *trpC* termination region was PCR-amplified with primers trpCt-1 and trpCt-2, leading to a fragment of 700 bp, using pAN7-1 as template (76). The *TetOn* system with a size of 2 kb was received by digestion of pJW123 (77) using the enzymes *Pme*I and *Hin*dIII. All three fragments were integrated via *Eco*RV into the pChS02 vector. The plasmid pChS03 contains a unique *Pme*I restriction site integrated by the oligonucleotide pyroA-3. Zinc cluster transcription factor encoding genes were PCR-amplified from genomic DNA of the *A. fumigatus* strain Af293. The received fragments were integrated via *Pme*I in the pChS03. Derived plasmids were checked by control digestion and sequencing.

### Construction of RFP-H2A fluorescence strains

The *H2A* gene was amplified from genomic DNA using the primers CS23/CS36 to receive a 936-bp fragment. The RFP was amplified using the primer pair CS35/CS22. We used genomic DNA of the *Tet-RFP* strain as template. Both fragments were integrated via *Pme*I in pME3856 (78). The received plasmid was ectopically integrated in AfS35, ACS280, and ACS449. Selection was carried out on a phleomycin-containing medium (final conc. 20 µg/mL). The received strains were named ACS411, ACS471, and ACS472.

### Construction of zinc cluster transcription factor overexpression strains

A *pyroA* deletion strain was used as parental strain for the overexpression library, which was constructed as follows: the *pyroA* (AFUA_5G08090) deletion construct pSK381 was generated by assembling the corresponding 5′ and 3′ regions of 1.8 and 1.4 kb amplified with Sv389/399 and Sv400/401, respectively, separated by the hygromycin resistance-conferring, recyclable marker module of pSK342 (79) via directional *Sfi*I-mediated ligation (80, 81) in the pCR blunt II TOPO vector (Invitrogen, Waltham, USA). From the resulting plasmid, a 6.8-kb gene replacement cassette was released by *Kpn*I/*Not*I digest and integrated via homologous recombination in the Δ*akuA* strainAfS35 (82) to obtain strain ACS01. Selection was carried out on minimal medium with pyridoxine (0.0001% final conc.) and hygromycin in a final concentration of 200 µg/mL. Clones were verified by Southern hybridization. Marker recycling was done in accordance with the protocol published by Krappmann et al. (79). The obtained *pyroA* deletion strain without resistance cassette was named ACS02.

Overexpression strains of the zinc cluster transcription factor encoding genes were constructed by integration of the different *TetOn* constructs via homologous recombination at the *pyroA* locus. The required fragments were received by digestion of the equivalent plasmid with *Hpa*I, *Psi*I, *Ssp*I, or *Nhe*I. Selection was performed on minimal medium without pyridoxine. All strains were validated by Southern hybridization. The Af293 was used as template for the transcription factors. For screening experiments two independent transformants of each overexpression strain were used. A *TetOn-RFP* (83) strain was used as control. This strain expresses an RFP derivate instead of a zinc cluster transcription factor. The according plasmid was constructed by using the primers RFP-1 and RFP-5. The pmCherry vector (Clontech, Mountain View, USA) was used as template. The received 0.7-kb fragment was cloned into pChS3 via *Pme*I.

### Construction of a *Tet-abcD*, *Tet-mdr1*, *Tet-atrI*, *Tet-abc3*, *and Tet-abc5* overexpression strains

The overexpression strains were constructed as follows: the primers abcD-1/abcD-2, mdr1-15/mdr1-16, atrI-1/atrI-2, abc1-1/abc1-2, and abc3-1/abc3-2 were used for amplification. The received fragments had sizes of 4.1, 4.2, 4.5, 4.4, and 5.2 kb. The AfS35 was used as template. The fragments were integrated into pChS3 via *Pme*I. The received plasmids were named pChS450, pChS452, and pChS451, pChS444, and pChS445. For transformation, the plasmids were digested with *Hpa*I, and the received fragments were integrated by homologous recombination at the *pyroA* locus of the Δ*pyroA* deletion strain ACS02. Selection was on minimal medium without pyridoxine. Transformants were verified for correctness by Southern hybridization.

### Construction of a *Tet-odrA-GFP* and *Tet-odrA*^D371A^*-GFP*

The *odrA* fragment was received by using the primers zcf46-1 and zcf46-14. The *odrA*^D371A^ fragment was obtained with the primers zcf46-1/zcf46-38 and zcf46-37/zcf46-14. We used the Af293 wild type as template. The GFP was received using the primers GFP-1 and GFP-4. We used the plasmid pME4435 as template (83). All fragments were integrated via *Pme*I in pChS3. The plasmids were named pChS271 (*Tet-odrA-GFP*) and pChS446 (*Tet-odrA*^D371A^-*GFP*) and digested with *Hpa*I. The received 10.1-kb fragment was integrated via homologous recombination at the *pyroA* locus of the Δ*pyroA* deletion strain ACS02. The received strains were named ACS280 and ACS449, respectively.

### Construction of knockout strains

Genomic DNA of the wild-type strain AfS35 was used as template for all deletion constructs. The deletion construct of *odrA* was generated as follows: the oligonucleotides zcf46-7 and zcf46-8 were used for amplification of the 5′UTR of *odrA* (1.5 kb), and the primers zcf46-9 and zcf46-10 were used for the 3′UTR (1.8 kb). The recyclable pyrithiamine resistance cassette published by Hartmann et al. was used as selection marker (84). The 5.3-kb fragment of this marker was received by digestion of plasmid pSK485 (84) with *Sfi*I. All fragments were integrated via *Eco*RV in the pBluescript II KS (+).

The construction of the *mdr1* deletion cassette was carried out as described for the Δ*mtrA* construct. The primers mdr1-1 and mdr1-2 were used for amplification to receive a 1.3-kb fragment upstream of the *mdr1* coding region. The primers mdr1-3 and mdr1-4 were used to amplify a 1-kb fragment of the 3′UTR.

The generated plasmids were digested with *Pme*I to receive fragments of 8.7 kb (*mtrA* deletion) and 7.7 kb (*mdr1* deletion), respectively, and integrated by homologous recombinatiom in the AfS35 (*odrA* deletion construct) and in the *Tet-odrA* strain (*mdr1* deletion construct), respectively. Selection was carried out on minimal medium containing pyrithiamine. For marker recycling, the protocol of Hartmann et al. was used with one modification (84). Xylose (0.5%) and glucose (0.5%) were added into the medium instead of 1% xylose. All strains were analyzed by Southern hybridization.

### Construction of a Δ*odrA* complementation strain with and without *GFP*, and the *odrA*^D371A^ strain

The *odrA* deletion construct (pChS242) was digested with *Swa*I. The oligonucleotides zcf46-13 and zcf46-25 were used to amplify *odrA*. To receive the mutated *odrA*^D371A^, the primers zcf46-13/zcf46-38 and zcf46-37/zcf46-25 were used. We used the Af293 as template. The oligonucleotides trpCt-4/trpCt-8 were used to amplify the termination region. Integration was carried out into the *odrA* deletion construct via the *Swa*I restriction site to receive pChS309 and pChS399, respectively. The pAN7-1 was used as template to amplify the *trpC* termination region. The plasmids were digested with *Pme*I. The obtained fragments (11.9 kb) were integrated by homologous recombination in the Δ*odrA* deletion strain. The obtained strains were named ACS352 (*odrA*) and ACS389 (*odrA*^D371A^). The complementation with GFP was carried out as follows: the *odrA* gene was received using the primers zcf46-13/zcf46-14. The GFP gene was amplified using the primers GFP-1 and GFP-4. We used the plasmid pME4435 as template (83). The pAN7-1 was used as template to amplify the *trpC* termination region. All fragments were integrated via *Swa*I in pChS242. The received plasmid was named pChS250. pChS250 was digested with *Pme*I to receive a 12.6-kb fragment, which was integrated via homologous recombination in the Δ*odrA* deletion strain. The received strain was named ACS81. Selection was carried out on minimal plates containing pyrithiamine. Transformants were streaked out on minimal medium with xylose (0.5%) and glucose (0.5%) for excision of the pyrithiamine resistance cassette. All strains were verified by Southern hybridization.

### RNA isolation and qPCR experiments

*A. fumigatus* was inoculated with 2 × 10^6^ spores/mL in 100-mL liquid minimal medium and incubated for 18 hours at 37°C while shaking. Received mycelium was shifted to fresh medium in the presence and absence of drugs or oxidative stress and incubated at 37°C for an additional 4 hours while shaking. For overexpression experiments, doxycycline was added in a final concentration of 50 µg/mL. RNeasy Plant Mini Kit (Qiagen, Hilden, Germany) was used according to the user’s manual. cDNA was generated using the QuantiTect Reverse Transcription Kit (Qiagen) according to the user’s manual. Total RNA was used as template. qRT PCR experiments were performed in a Bio-Rad CFX Connect Real-Time System (Bio-Rad Laboratories Inc., Hercules, USA) using the MESA GREEN qPCR kit for SYBR Assay (Eurogentec, Lüttich, Belgium) according to the user’s manual. For normalization, the *H2A* and the *eIF2B* gene were used. Experiments were carried out with three or four biological replicates except for the validation of the overexpression library (one biological replicate) and the RNA-seq data (two biological replicates). All genes were tested in technical triplicates.

### Genome-wide transcriptional analysis (RNA-seq)

Total RNA was received from submerged cultures using the RNeasy Plant Mini Kit (Qiagen) according to the user’s manual. RNA sequencing was performed as described by Thieme et al. (85) at the Core Unit, the Transcriptome and Genome Analysis Laboratory, University Medical Center Göttingen. Three biological replicates were used.

Raw reads were aligned against the *Aspergillus fumigatus* genome Af293 (Aspergillus_fumigatus.ASM265v1.42). For alignment, the HISAT2 was used. The FungiDB (42) was used for classification of the different genes. For significance, the adjusted *P* value was used.

### Chromatin immunoprecipitation

Chromatin immunoprecipitation (ChIP) experiments were carried out using the *Tet-odrA-GFP* and the *odrA-GFP* strain. We used the AfGB76 (83) (GFP overexpressing strain) and the wild type as controls. For each of these strains at least three independent biological replicates were prepared. The base of the ChIP experiment, performed for this study, was the protocol described by references (86, 86), with appropriate modifications tailored for *A. fumigatus*. In short, for the ChIP-seq experiment, 2-L flasks containing 500-mL liquid minimal medium were inoculated with 2 × 10^6^ spores/mL. Cultures were grown overnight under shaking at 37°C. For induction, 50 µg/mL of doxycycline was added for an additional 4 hours. For the ChIP experiments with the *odrA-GFP* expressed under its native promoter, the same setup was used as mentioned before but without adding doxycycline. Mycelia were collected, dried shortly on paper and weighted to have the same initial mass. The mycelia were then cross-linked in 1% formaldehyde solution for 15 minutes under vacuum and at room temperature. Fixation was stopped by the addition of 0.125 M glycine under vacuum for another 5 minutes. Next, the cross-linked mycelia were washed with washing buffer (86) and then dried on paper and subsequently frozen in liquid nitrogen. Following the pulverization of the samples, nuclei were isolated based on the protocol of Kaufmann et al. (86), and chromatin was sheared using Covaris M220 Focused-ultrasonicator (to approximately an average of 500-bp fragments). It followed the immunoprecipitation by using the anti-GFP antibody/ChIP Grade (ab290, dilution 1:300; Abcam, Cambridge, UK) and then the Protein A/G PLUS-agarose beads (Santa Cruz Biotechnology, Dallas, USA). Beads were washed with immunoprecipitation (IP) buffer (86) and treated with proteinase K/recombinant PCR grade (Roche Diagnostics GmbH, Basel, Switzerland) for reverse cross-linking. The ChIPed DNA then purified by using the MiniElute PCR purification kit (Qiagen) and subsequently used in ChIP qPCRs to investigate the binding of the OdrA-GFP into certain regions upstream from the ATG of the corresponding genes by using the SsoAdvance Universal SYBR Green Supermix (Bio-Rad Laboratories Inc.) and the cycler CFX Connect Real-Time System (Bio-Rad Laboratories Inc.). Specific sets of primers were used for analysis of the putative binding regions (Table S9). ChIP-seq libraries and sequencing were performed at the NGS Integrative Genomics Core Unit, University Medical Center Göttingen. Briefly, quality and quantity of IP-DNA were assessed using a fragment analyzer. Libraries were constructed using the TruSeq ChIP Sample Preparation kit (Illumina, San Diego, USA) with minor adaptations and sequenced on the HiSeq 4,000 platform (Illumina) in single read sequencing mode of 50 bp, which yielded an average of 40 Mio reads per sample. Sequence images were transformed with Illumina software Base-Caller to BCL files, which was demultiplexed to fastq files with bcl2fastq (version 2.20) generating a FastQC for data quality control.

### ChIP-seq analysis

The subsequent analysis of the produced ChIP-seq data was performed with the GALAXY platform (87), within the Galaxy BLUM @ LAFUGA server, Gene Center, Ludwig Maximilian University of Munich. In short, the raw reads from the sequencing were mapped against the *Aspergillus fumigatus* genome (downloaded from fungidb.org: FungiDB-48_AfumigatusAf293_Genome.fasta) using Bowtie2 (Galaxy version 2.3.4.2) (88). The produced mapped files were then used in the tool MACS2 (Galaxy version 2.1.0–6) (89) to identify statistically significant (*P* value of <0.05) peaks among different biological replicates of OE-*GFP* and *Tet-odrA-GFP* samples. MEME-ChIP tool (Galaxy version 2.1.0–6) (90) was used for *de novo* motif discovery, as input sequences of 100 bp that lie below the summit of the 150 top-scored peaks were used, identified by MACS2, from each set of analysis. ChIP-seq peaks were visualized in a genome browser of Integrated Genome Broswer (91). To examine the distribution of the ChIP-seq peaks, identified by MACS2, over different genomic features, the R-package of ChIPseeker (version 1.26.2) (92) was utilized via the R-studio console (93), provided and maintained by the Gesellschaft für Wissenschaftliche Datenverarbeitung GmbH Göttingen. For generating Venn diagrams, the InteractiVenn tool was used (94). The processing of the corresponding figure (Fig. 4) was done by the vector-graphics editor Inscape (Inkscape Project, 2020; Inkscape, available at https://inkscape.org).

### Protein extraction and western experiments

Freshly harvested spores (2 × 10^6^ spores/mL) were inoculated in 100-mL minimal medium and grown for 18 hours. Received cultures were shifted to fresh medium in the presence and absence of 50-µg/mL doxycycline and incubated for an additional 4 hours. We used the AfS35 wild type negative control. Mycelium was harvested and washed with 0.96% sodium chloride solution and grinded in liquid nitrogen. Approximately 0.5 mL of grinded mycelium was mixed with 500 µL of B buffer (100 mM Tris-HCl, pH 7.2, 200 mM NaCl, 20% glycerol, 5 mM EDTA, freshly added β-mercaptoethanol in a final concentration of 14.3 mM, Complete Protease Inhibitor Cocktail Tablets [Roche Diagnostics GmbH]) according to the user’ s manual. The received suspension was centrifuged at 13,000 rpm for 10 minutes at 4°C. Same amounts of proteins were loaded on a 12% SDS-PAGE. Afterward, proteins were transferred to a nitrocellulose membrane (Merck, Darmstadt, Germany). The enhanced chemiluminescence method was used for detection as previously described (95). Green fluorescent protein (GFP) was detected using the α-GFP antibody (sc-9996, Santa Cruz Biotechnology). We used the horseradish peroxidase-coupled anti-mouse (115-035-003; Jackson Immuno Research, West Grove, USA) as secondary antibody. For detection, we mixed and incubated a luminol solution (2.5 mM Luminol, 400 µM Paracoumarat, 100 mM Tris-HCl, pH 8.5) and hydrogen peroxide solution (5.4 mM H_2_O_2_, 100 mM Tris-HCl, pH 8.5) with the membrane for approximately 2 minutes. Afterward, the membrane was exposed to an Amersham Hyperfilm ECL (GE Healthcare Life Technologies) for approximately 1 minute and developed.

### Growth rate experiments

Strains were grown on malt extract agar (96) for 5 days at 37°C. *A. fumigatus* spores were harvested and diluted in phosphate-buffered saline (PBS)-Tween to a final concentration of 2,000 spores/µL and stored at 4°C for up to 5 days. For the 96-well plate growth assay, 20-µL spore suspension was added to 180-µL minimal medium containing doxycycline (Sigma-Aldrich, St Louis, USA) at a final concentration of 50 µg/mL for the induction of the *tet* promoter. Growth was monitored by OD600 nm readout in a robotic incubator Cytomat42 (Cytomat42, Thermo Fisher Scientific) combined with a robotic arm (Rackrunner; Hamilton Bonaduz AG, Bonaduz, Switzerland) and measured in a Synergy H1 plate reader (BioTek Instruments Inc., Winooski, VT, USA) in 2-hour intervals for 36 hours. Three technical replicates of at least two biological replicates were performed. The *Tet-RFP* strain was used as reference. Growth curves were analyzed with the “growthcurver” package of the statistic software R (97, 98).

### Microscopy experiments

Approximately 500 spores of the tested strains were incubated overnight at 37°C on a cover slide in the presence and absence of 5- or 50-µg/mL doxycycline. Drugs were added for an additional 4 hours in the following concentrations: amphotericin B 0.65 µg/mL, voriconazole 0.65 mg/mL, itraconazole 5 µg/mL, and menadione 0.04 mM. Received samples were used for microscopy using a confocal Zeiss Observer Z.1 microscope (ZEISS, Oberkochen, Germany) including a CSU-X1 A1 confocal scanner unit (Yokogawa, Ratingen, Germany), QuantEM:512SC digital camera (Photometrics, Tucson, AZ, USA), and SlideBook (version 6.0) software package (Intelligent Imaging Innovations, Göttingen, Germany).

### Spot test assays and screening of the overexpression library

Freshly harvested spores were adjusted to a concentration of 10^6^ spores/mL in sodium chloride with Tween 20 (0.96% NaCl and 0.02% Tween 20). Approximately 2,000 spores were spotted on MM. For induction of the overexpression strains, we added 50-µg/mL doxycycline. We added amphotericin B with a final concertation of 0.75 or 1.0 µg/mL and voriconazole with a final concentration of 0.5 µg/mL or 0.75 µg/mL as drugs. Two independent replicates were analyzed in the screen. We used the *Tet-RFP* strain as control. Plates were incubated for 3 days at 37°C. Candidates showing an overexpression phenotype in comparison to the control were tested in a second independent experiment to avoid false positives. For determination of the colony size, we measured the diameters of the colonies and determined the quotient between overexpression strain and control strain (*zcf/Tet-RFP*). Strains were categorized as “reduced growth” when the average of the two quotients was ≤0.9 (Table S9).

### Dilution assays

Dilution spot tests were carried out as described by Dichtl et al. (77) with modifications. MM was used instead of complete medium. Plates without drugs were incubated for two days. In the presence of antifungal drugs, the incubation was prolonged up to 3 days at 37°C. Amphotericin B in final concentrations of 0.75 and 1.25 µg/mL, voriconazole in final concentrations of 0.65 and 1.0 µg/mL, and itraconazole in final concentrations of 4 µg/mL and 5 µg/mL, respectively, were used as drugs. To induce oxidative stress, menadione was added in a final concentration of 0.015 mM. For induction of the *TetOn* system, 50-µg/mL doxycycline was added. Two independent experiments were carried out.

### MIC tests

Approximately 2,000 fresh harvested spores were used for MIC assays. We used voriconazole, amphotericin B, and itraconazole as antifungal drugs. Spores were incubated in minimal medium in a 96-well plate for 48 hours at 37°C. The MIC value was determined by the first well without any growth of the fungus. Three independent experiments were carried out.

## Data Availability

All raw data have been deposited at NCBI [BioProject accession numbers PRJNA875024 (ChIP-seq data set) and PRJNA874944 (RNA-seq data set)].

## SUPPLEMENTAL MATERIAL

Fig. S1 mbio.02628-23-s0001.pdf Scheme of the construction of the overexpression library.

Fig. S2 mbio.02628-23-s0002.pdf Construction of the ∆*pyroA* strain and the Tet-RFP control strain.

Fig. S3 mbio.02628-23-s0003.pdf *A. fumigatus* phenotypes caused by overexpression of zinc cluster transcription factor encoding *zcf* genes.

Fig. S4 mbio.02628-23-s0004.pdf Validation of the overexpression library.

Fig. S5 mbio.02628-23-s0005.pdf Transcription factors which lead to an altered phenotype when overexpressed.

Fig. S6 mbio.02628-23-s0006.pdf Lag phase delay caused by the overexpression of *zcf* genes, providing drug tolerance to *A. fumigatus*, is an indicator for reduced fitness during vegetative growth.

Fig. S7 mbio.02628-23-s0007.pdf Validation of the *odrA*/*mdu2* RNA-seq data and investigation of different transporter genes on drug resistance.

Fig. S8 mbio.02628-23-s0008.pdf OdrA binds to the promoter of mdr1 in a *Tet-odrA-GFP* strain.

Fig. S9 mbio.02628-23-s0009.pdf Influence of increased nuclear accumulation of OdrA/Mdu2 on drug response.

Supplemental tables mbio.02628-23-s0010.xlsx Tables S1-S10.

ASM does not own the copyrights to Supplemental Material that may be linked to, or accessed through, an article. The authors have granted ASM a non-exclusive, world-wide license to publish the Supplemental Material files. Please contact the corresponding author directly for reuse.
