# Supplementary material for: Induction of Aspergillus fumigatus zinc cluster transcription factor OdrA/Mdu2 provides combined cellular responses for oxidative stress protection and multiple antifungal drug resistance
Source: mBio. 2023 Nov 20;14(6):e02628-23. doi: 10.1128/mbio.02628-23 (PMC10746196; doi:10.1128/mbio.02628-23)
Supplement: Fig. S1 — Scheme of the construction of the overexpression library. [file mbio.02628-23-s0001.pdf]

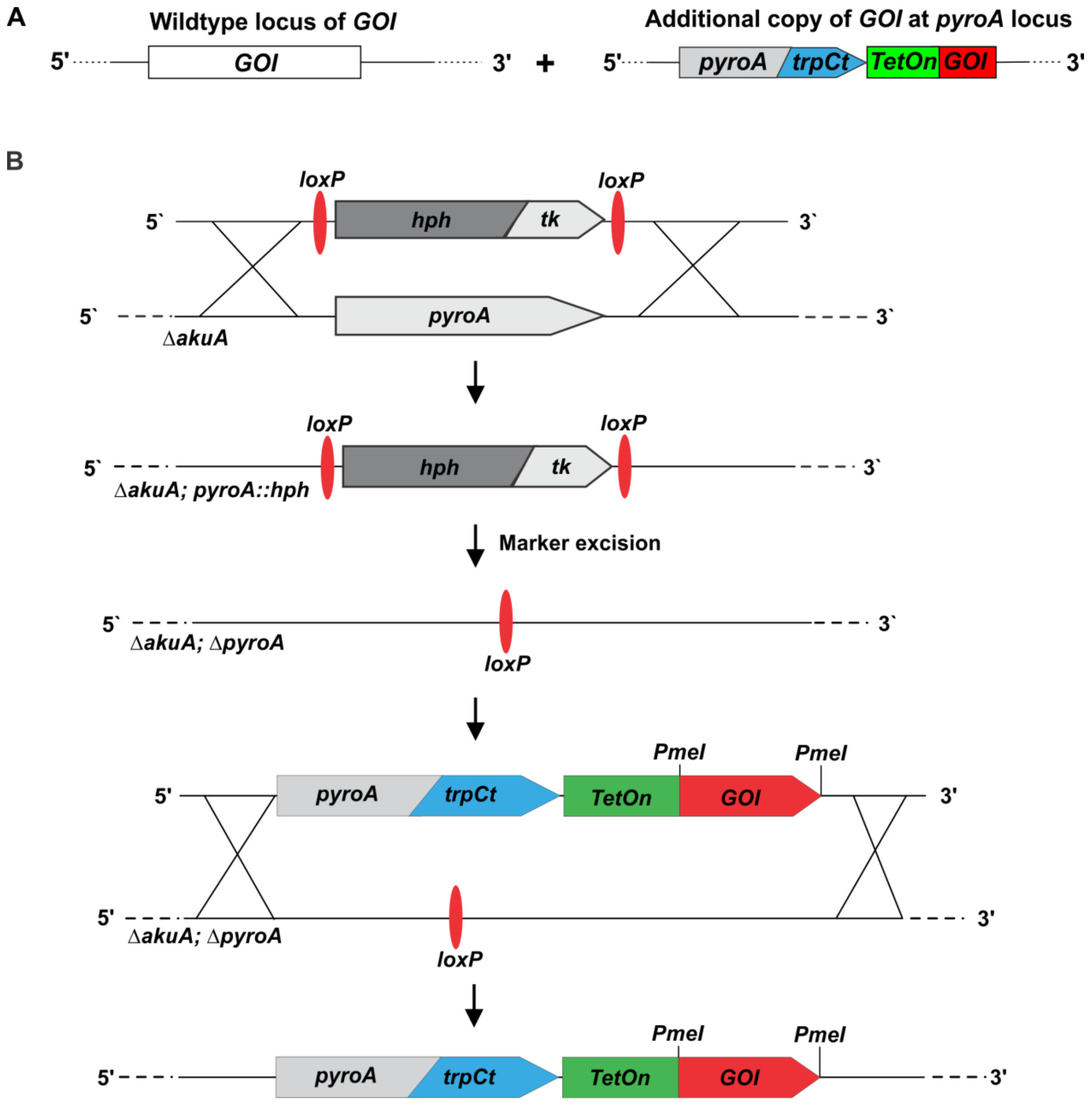

S1 Fig: Scheme of the construction of the overexpression library. (A) Scheme of the construction of the *zcf* overexpression library using the *A. fumigatus* adapted *TetOn* system (74). The inducible *TetOn* promoter with the *zcf* gene of interest was integrated at the *pyroA* locus. The integrated *pyroA* gene was fused to the *trpC* termination region (*trpCt*). The overexpression strains contain the wildtype allele and an additional copy of the *zcf* gene of interest (*GOI*), which can by induced by doxycycline. (B) Detailed scheme of the construction of the *zcf* overexpression. A *pyroA* deletion strain was used as parental strain for the overexpression library. This strain was constructed by using the hygromycin resistance cassette (*hph*) as selection marker, a thymidine kinase (*tk*) for counter-selection, and *loxP* sites for the marker excision. Excision of the resistance cassette was induced by using a Cre-lox recombinase. The overexpression constructs consist of the *pyroA* gene (*pyroA*), a *trpC* terminator (*trpCt*), the *TetOn* system (*TetOn*) and the *GOI*. The constructs were flanked by the 5'UTR and the 3'UTR of the *pyroA* gene, respectively. Constructs were integrated *via* homologous recombination at the Δ*pyroA* locus. As selection marker the *pyroA* gene was used.
