# Supplementary material for: Induction of Aspergillus fumigatus zinc cluster transcription factor OdrA/Mdu2 provides combined cellular responses for oxidative stress protection and multiple antifungal drug resistance
Source: mBio. 2023 Nov 20;14(6):e02628-23. doi: 10.1128/mbio.02628-23 (PMC10746196; doi:10.1128/mbio.02628-23)
Supplement: Fig. S2 — Construction of the ∆pyroA strain and the Tet-RFP control strain. [file mbio.02628-23-s0002.pdf]

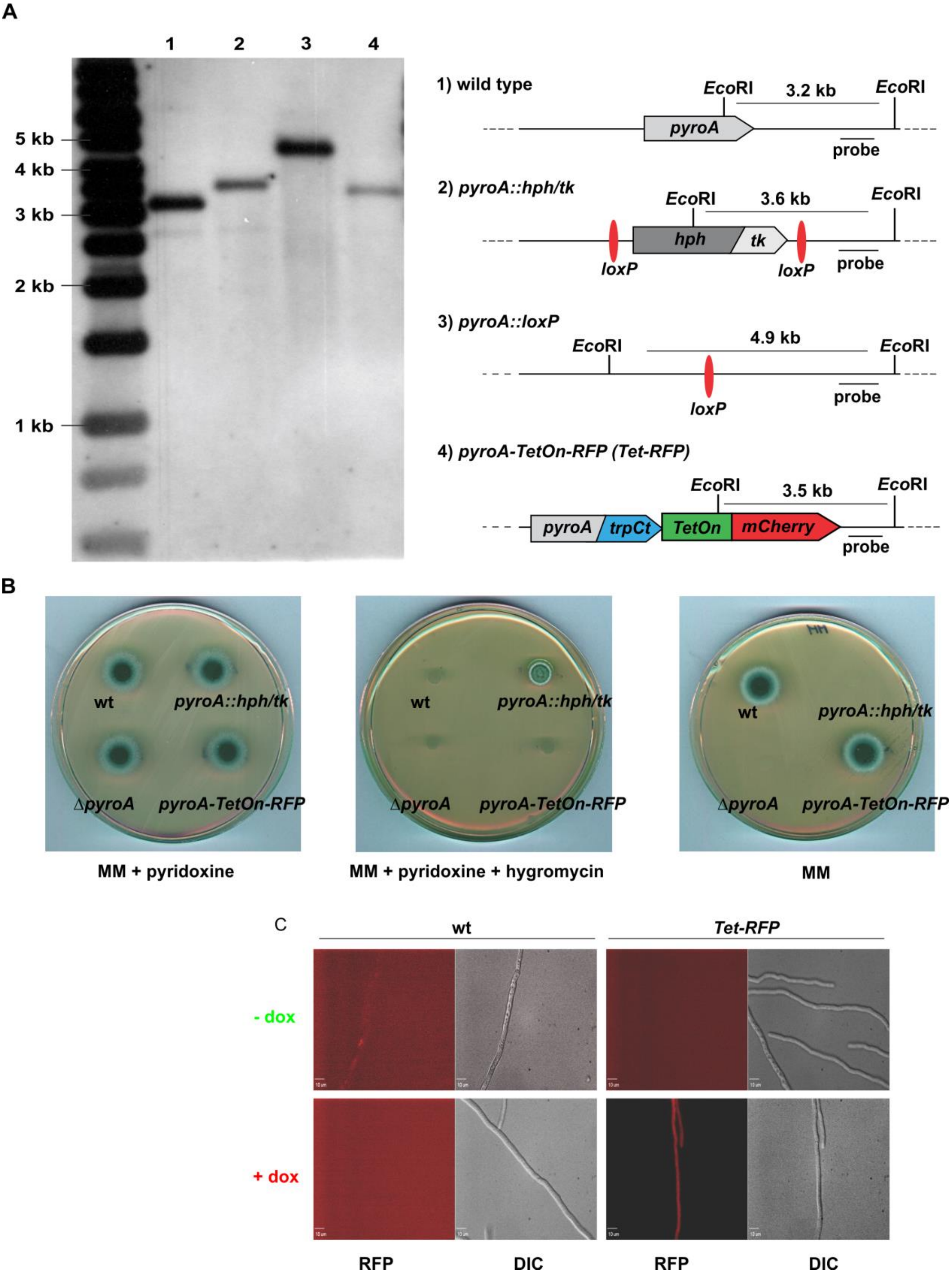

S2 Fig: Construction of the Δ*pyroA* strain and the *Tet-RFP* control strain. (A) Southern hybridization experiments to confirm the deletion of *pyroA* and the integration of a *TetOn-RFP* construct. The used probe binds in the 3' region. Strains were verified before and after marker excision. *Eco*RI was used as restriction enzyme. (B) Spot-test of the *pyroA* deletion strain with and without hygromycin resistance cassette, the wildtype and the *pyroA-Tet-RFP* strain. MM plates with and without hygromycin/pyridoxine were used. Approx. 2000 spores were spotted. Plates were incubated for 3 days at 37°C. (C) The *Tet-RFP* strain and the wildtype were grown on cover slides with and without 50 µg/ml doxycycline. Samples were grown over-night at 37°C. Microscopy was performed in transmitted light

(DIC) and by using the S561R filter (illumination at ~588 nm) for red fluorescence (RFP). Red fluorescence was only observed for the *Tet-RFP* strain in presence of doxycycline. Scale bars represent 10 µm.
