# Supplementary material for: Induction of Aspergillus fumigatus zinc cluster transcription factor OdrA/Mdu2 provides combined cellular responses for oxidative stress protection and multiple antifungal drug resistance
Source: mBio. 2023 Nov 20;14(6):e02628-23. doi: 10.1128/mbio.02628-23 (PMC10746196; doi:10.1128/mbio.02628-23)
Supplement: Fig. S3 — A. fumigatus phenotypes caused by overexpression of zinc cluster transcription factor encoding zcf genes. [file mbio.02628-23-s0003.pdf]

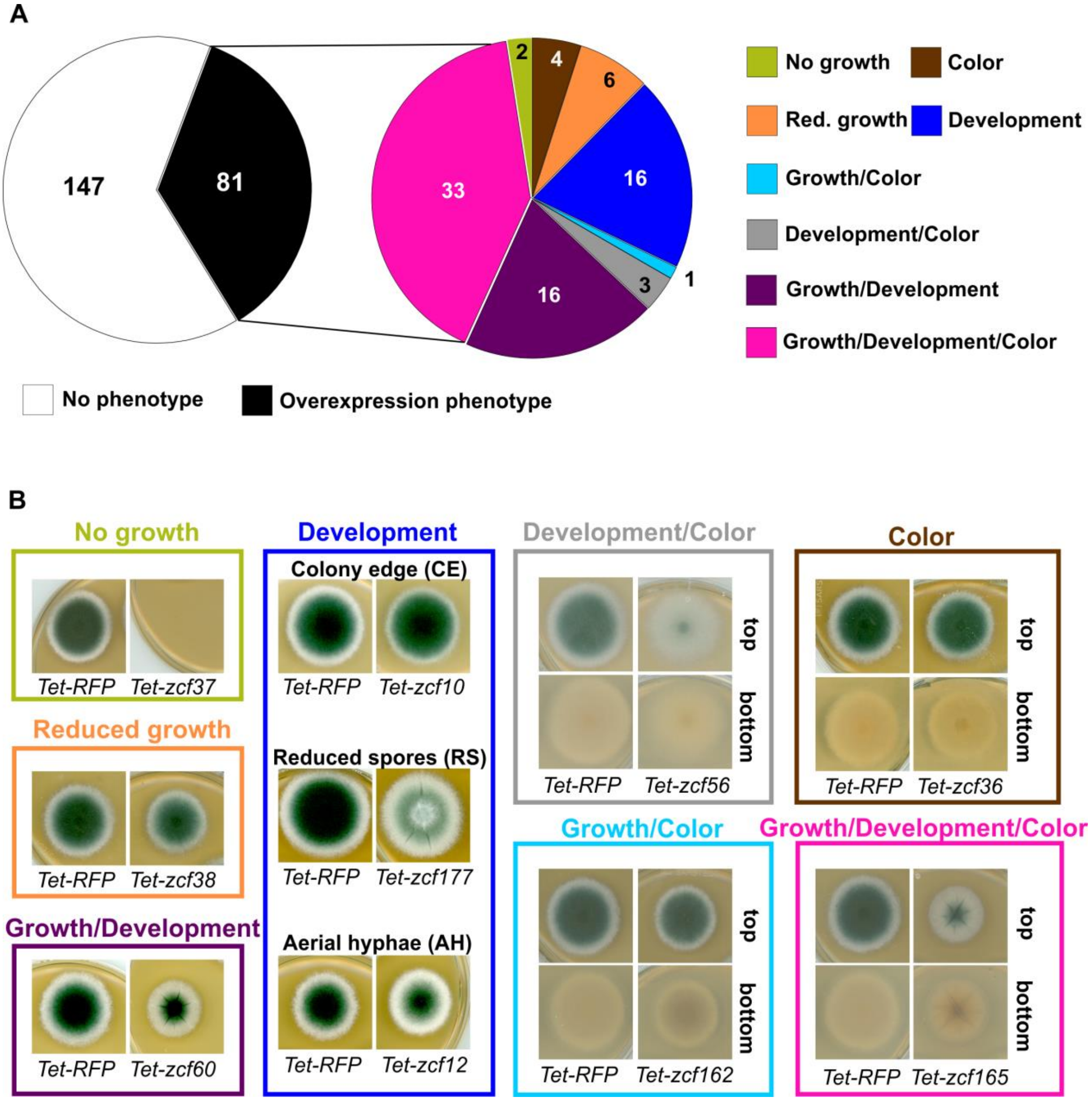

S3 Fig: *A. fumigatus* phenotypes caused by overexpression of zinc cluster transcription factor encoding *zcf* genes. (A) Pie charts of different overexpression phenotype classes of the 228 zinc cluster transcription factor genes. Left diagram shows the numbers of transcription factors with or without overexpression phenotype. The right pie chart depicts single or combined phenotypes of growth, development or colony color. (B) Examples for each class of phenotypes. As reference the control (*Tet-RFP*) strain is shown. Developmental phenotypes were separated in aerial hyphae production, changed colony edge or reduced number of conidiospores. Only for those strains leading to an altered colony color the bottom of the colony is shown as well. Overexpression of 81 (36%) out of the 228 investigated genes resulted in altered colony phenotypes on media lacking any additional drugs. The phenotypes were classified into four different categories. In cases where the classification was ambiguous the most prominent phenotype class was assigned. (i) Growth impairment as a sole phenotype ranged between no growth (green box) and reduced colony size (orange box). (ii) Distinct changes in development included less spores, increased aerial hyphae production or differences at the edge of the colony (blue box). (iii) Colorant production used as an indicator for a putatively dysregulated secondary metabolite production (brown box). (iv) Altered colony appearance featuring combinations as developmental changes combined with a growth defect (purple box) and triple phenotypical effects on growth, development as well as colorant production (pink box).
