# Supplementary material for: Induction of Aspergillus fumigatus zinc cluster transcription factor OdrA/Mdu2 provides combined cellular responses for oxidative stress protection and multiple antifungal drug resistance
Source: mBio. 2023 Nov 20;14(6):e02628-23. doi: 10.1128/mbio.02628-23 (PMC10746196; doi:10.1128/mbio.02628-23)
Supplement: Fig. S4 — Validation of the overexpression library. [file mbio.02628-23-s0004.pdf]

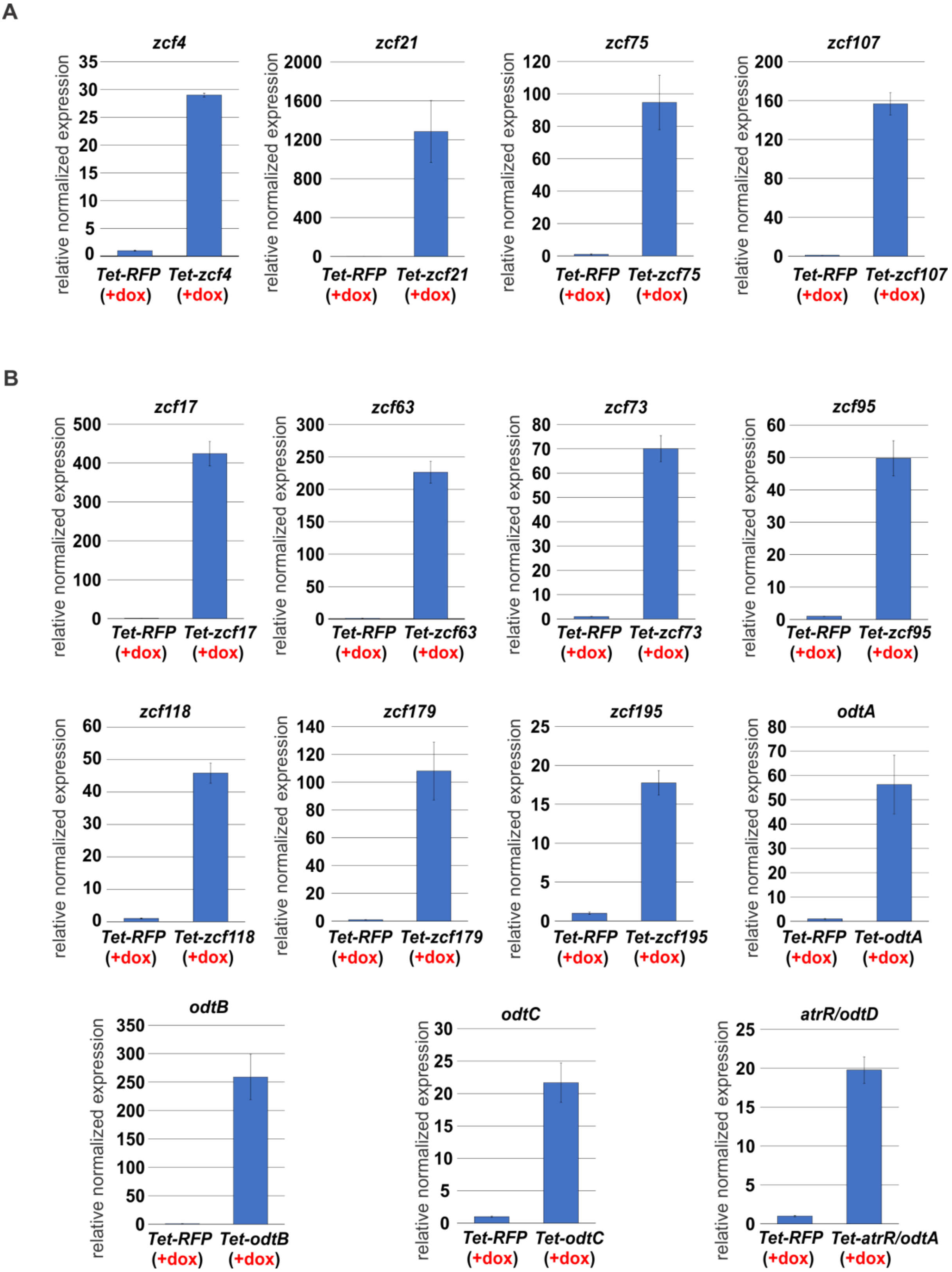

S4 Fig: Validation of the overexpression library. qPCR experiments of the *Tet-RFP* strain, four randomly chosen overexpression strains (A) and the eleven overexpression strains increasing drug tolerance (B). Strains were incubated for 18h in liquid MM and shifted to fresh medium in presence (+dox) of 50 µg/ml doxycycline for additional 4h. *H2A* was used for normalization. Levels for the *Tet-RFP* strain (*Tet-RFP*) were set to 1. Graph indicates mean ± standard errors from one experiment with three technical replicates.
