# Supplementary material for: Induction of Aspergillus fumigatus zinc cluster transcription factor OdrA/Mdu2 provides combined cellular responses for oxidative stress protection and multiple antifungal drug resistance
Source: mBio. 2023 Nov 20;14(6):e02628-23. doi: 10.1128/mbio.02628-23 (PMC10746196; doi:10.1128/mbio.02628-23)
Supplement: Fig. S5 — Transcription factors which lead to an altered phenotype when overexpressed. [file mbio.02628-23-s0005.pdf]

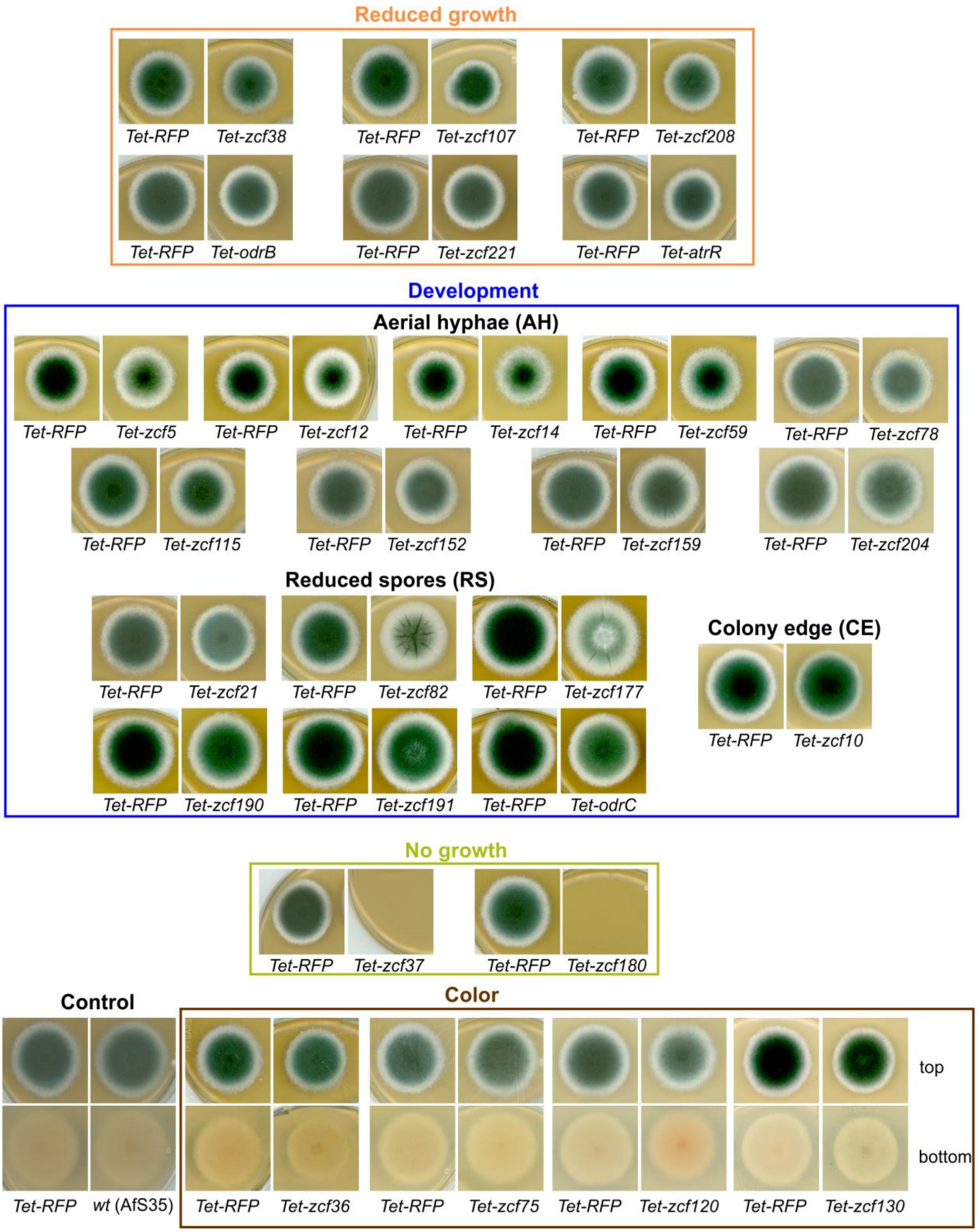
Reduced growth
Tet-RFP Tet-zcf38 Tet-RFP Tet-zcf107 Tet-RFP Tet-zcf208
Tet-RFP Tet-odrB Tet-RFP Tet-zcf221 Tet-RFP Tet-atrR
Development
Aerial hyphae (AH)
Tet-RFP Tet-zcf5 Tet-RFP Tet-zcf12 Tet-RFP Tet-zcf14 Tet-RFP Tet-zcf59 Tet-RFP Tet-zcf78
Tet-RFP Tet-zcf115 Tet-RFP Tet-zcf152 Tet-RFP Tet-zcf159 Tet-RFP Tet-zcf204
Reduced spores (RS)
Tet-RFP Tet-zcf21 Tet-RFP Tet-zcf82 Tet-RFP Tet-zcf177
Tet-RFP Tet-zcf190 Tet-RFP Tet-zcf191 Tet-RFP Tet-odrC
Colony edge (CE)
Tet-RFP Tet-zcf10
No growth
Tet-RFP Tet-zcf37 Tet-RFP Tet-zcf180
Control
Color
top
bottom
Tet-RFP wt (AfS35) Tet-RFP Tet-zcf36 Tet-RFP Tet-zcf75 Tet-RFP Tet-zcf120 Tet-RFP Tet-zcf130

Growth/Development

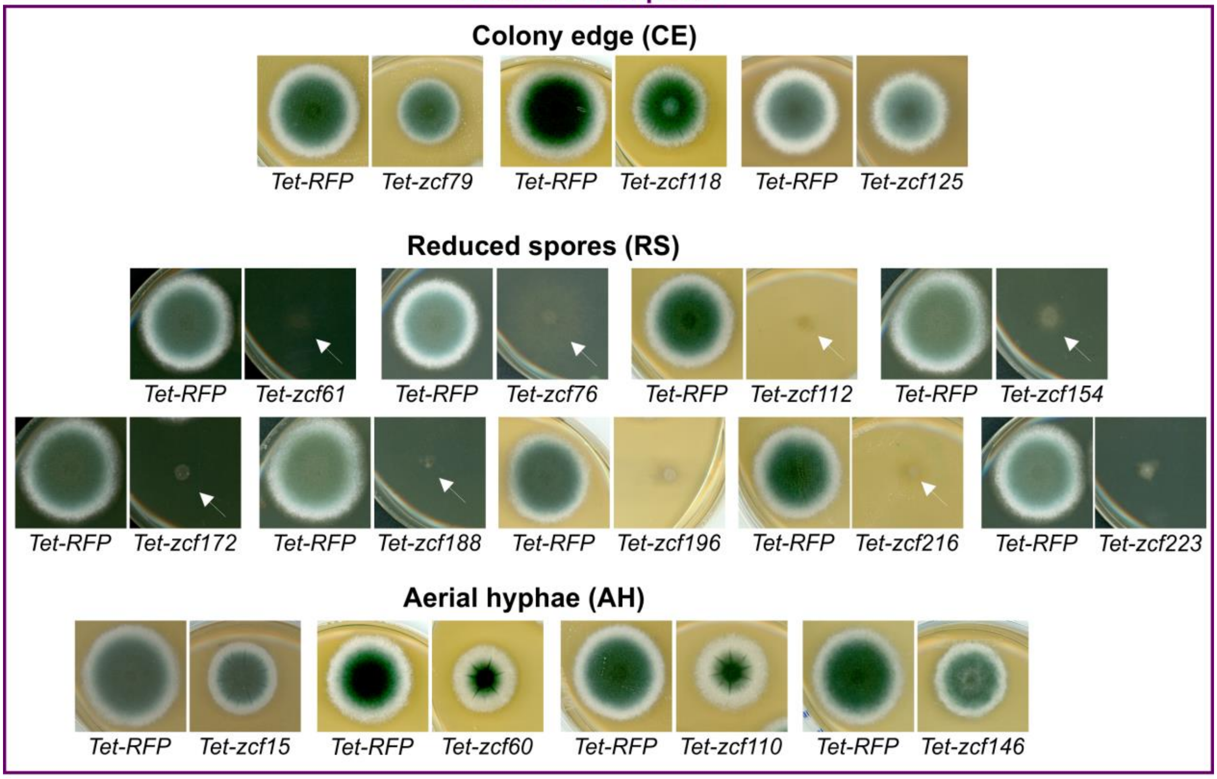

Development/Color

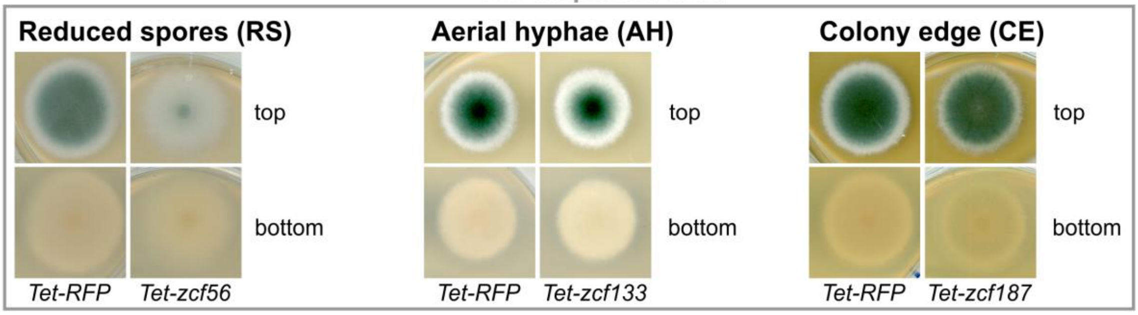

Growth/Color

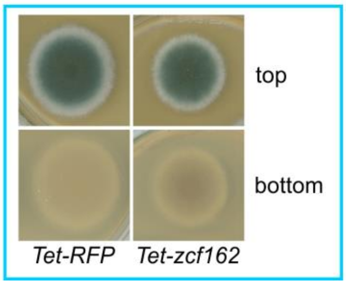

Growth/Development/Color

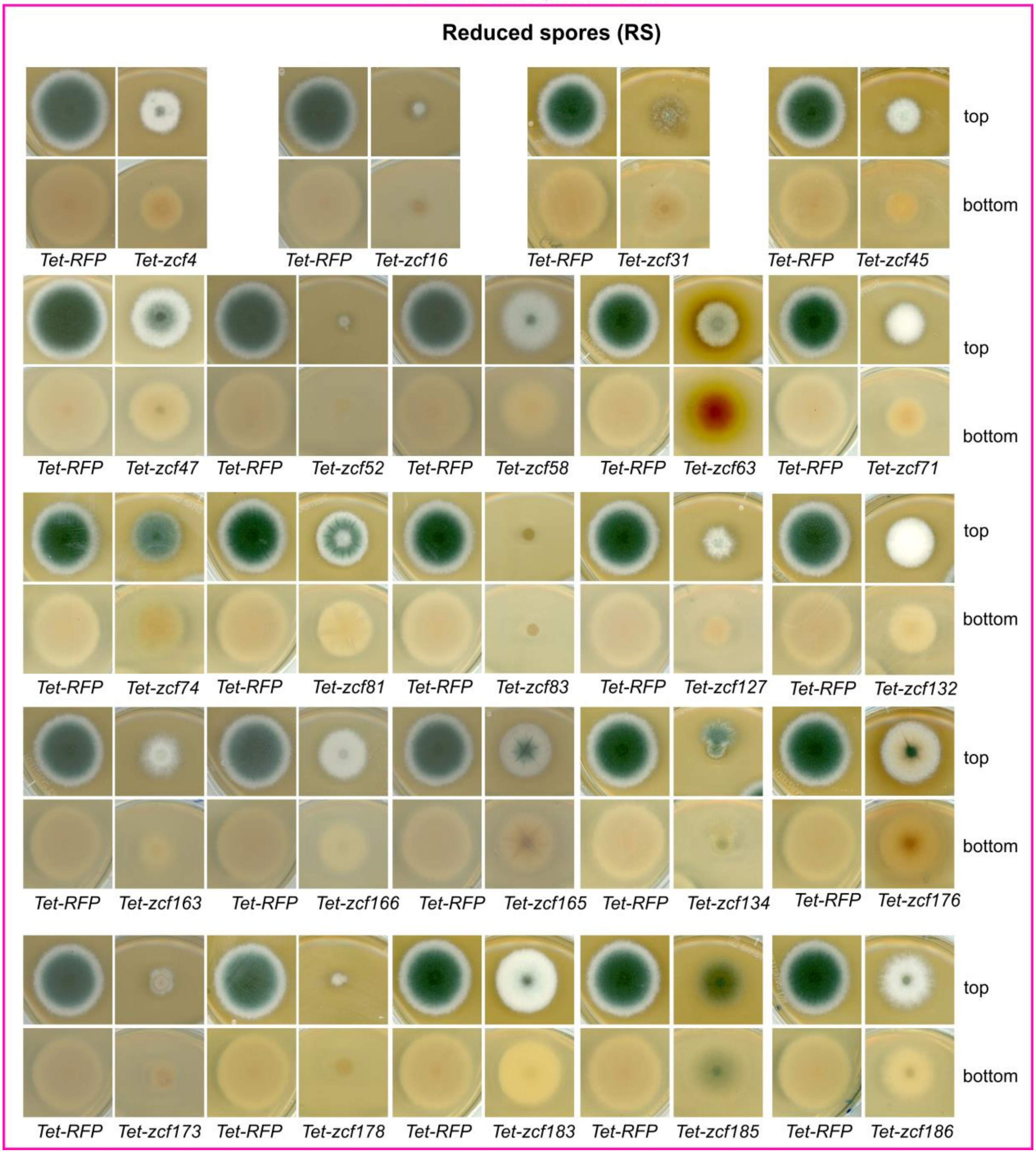

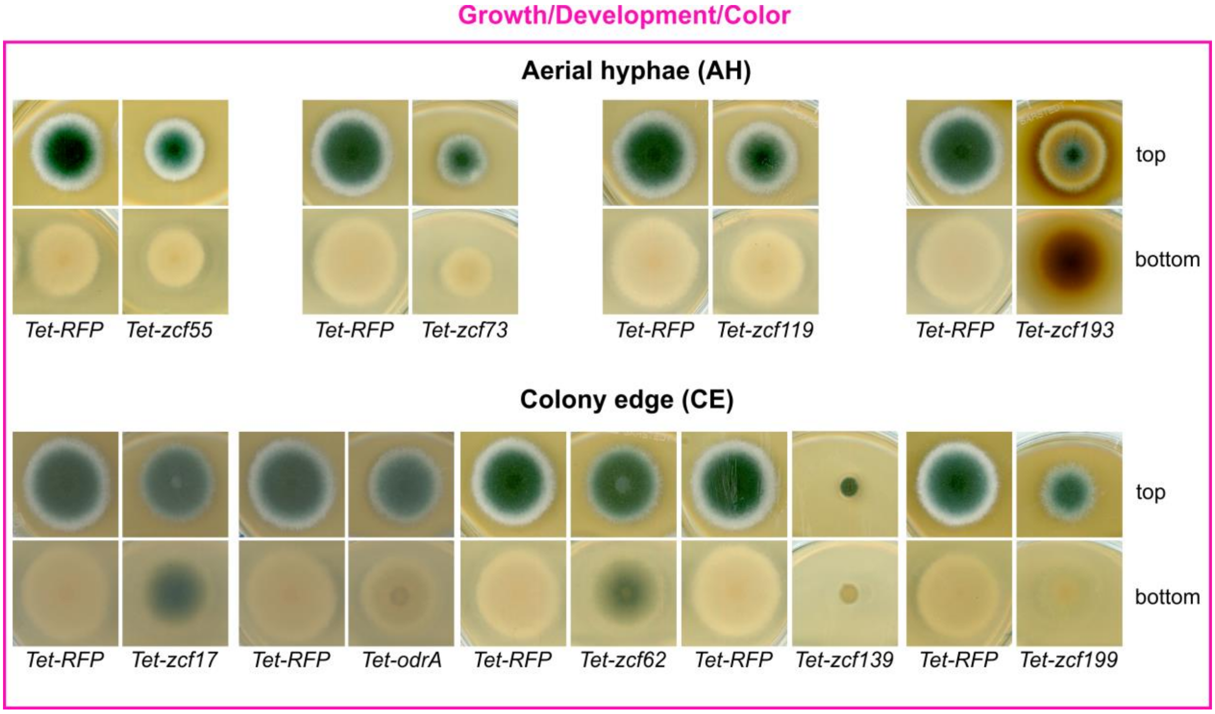

S5 Fig: Transcription factors which leads to an altered phenotype when overexpressed. Spot-tests of overexpression strains having a single phenotype, combinations of two different phenotypes, or a combination of three different phenotypes. The developmental phenotype is subdivided in aerial hyphae (AH), reduced spores (RS), and changed colony edge (CE). As reference the *Tet-RFP* and the wildtype were used. Approx. 2000 spores were spotted on MM in presence of 50 µg/ml doxycycline. Plates were incubated for three days at 37°C.
