# Supplementary material for: Induction of Aspergillus fumigatus zinc cluster transcription factor OdrA/Mdu2 provides combined cellular responses for oxidative stress protection and multiple antifungal drug resistance
Source: mBio. 2023 Nov 20;14(6):e02628-23. doi: 10.1128/mbio.02628-23 (PMC10746196; doi:10.1128/mbio.02628-23)
Supplement: Fig. S6 — Lag phase delay caused by the overexpression of zcf genes, providing drug tolerance to A. fumigatus, is an indicator for reduced fitness during vegetative growth. [file mbio.02628-23-s0006.pdf]

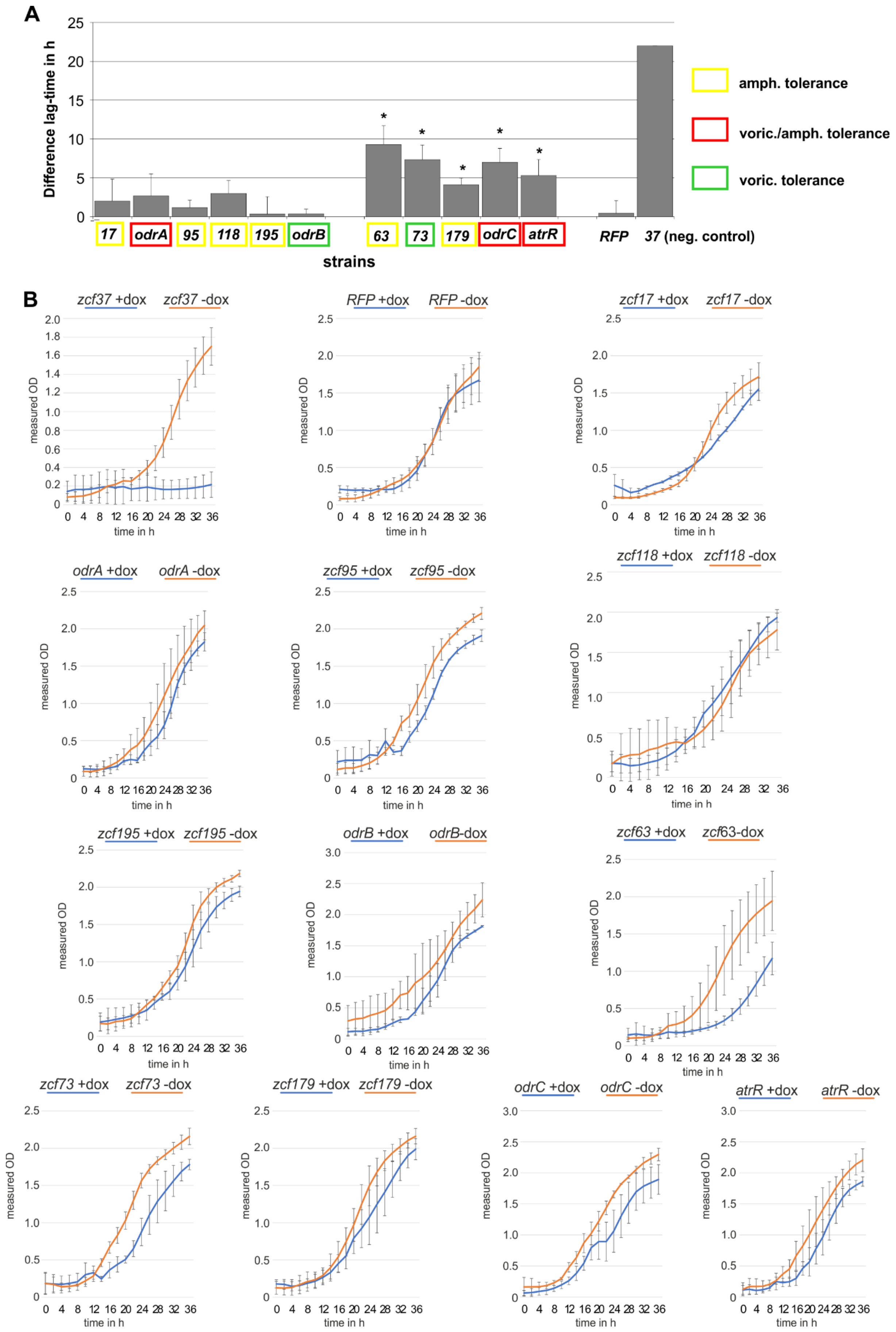

S6 Fig: Lag phase delay caused by the overexpression of *zcf* genes, providing drug tolerance to *A. fumigatus*, is an indicator for reduced fitness during vegetative growth. Fitness of the different

overexpression strains during vegetative growth was calculated by the lag-time difference between inducing (+dox) and non-inducing (-dox) conditions. Fitness of the strains was determined in presence and absence of doxycycline. The optical density was measured in 96 well plates in liquid medium. The onset of rapid growth or the end of lag phase was a reliable parameter for fitness. The end of the lag phase was defined as the time point when the optical density increases between successive measurements (2h interval) with an absorbance unit greater than 0.1. The later changes of optical density and further continuation of the growth was not analyzed further. (A) Bar diagram of the lag phase difference of the tested strains. The overexpression of *zcf63*, *zcf73*, *zcf179*, *odrC* and *atrR/odrD* show significantly reduced fitness. Significance was calculated using the student`s t-test with $p<0.05$ and indicated by asterisks. (B) Growth curves of the different *zcf* overexpression strains. Growth in liquid medium was measured every two hours. Incubation was with and without doxycycline. For all experiments, the *Tet-RFP* strain was used as reference. As negative control the *Tet-zcf37* overexpression strain was used (*zcf37*), which is unable to grow under inducing conditions.
