# Supplementary material for: Induction of Aspergillus fumigatus zinc cluster transcription factor OdrA/Mdu2 provides combined cellular responses for oxidative stress protection and multiple antifungal drug resistance
Source: mBio. 2023 Nov 20;14(6):e02628-23. doi: 10.1128/mbio.02628-23 (PMC10746196; doi:10.1128/mbio.02628-23)
Supplement: Fig. S7 — Validation of the odrA/mdu2 RNA-seq data and investigation of different transporter genes on drug resistance. [file mbio.02628-23-s0007.pdf]

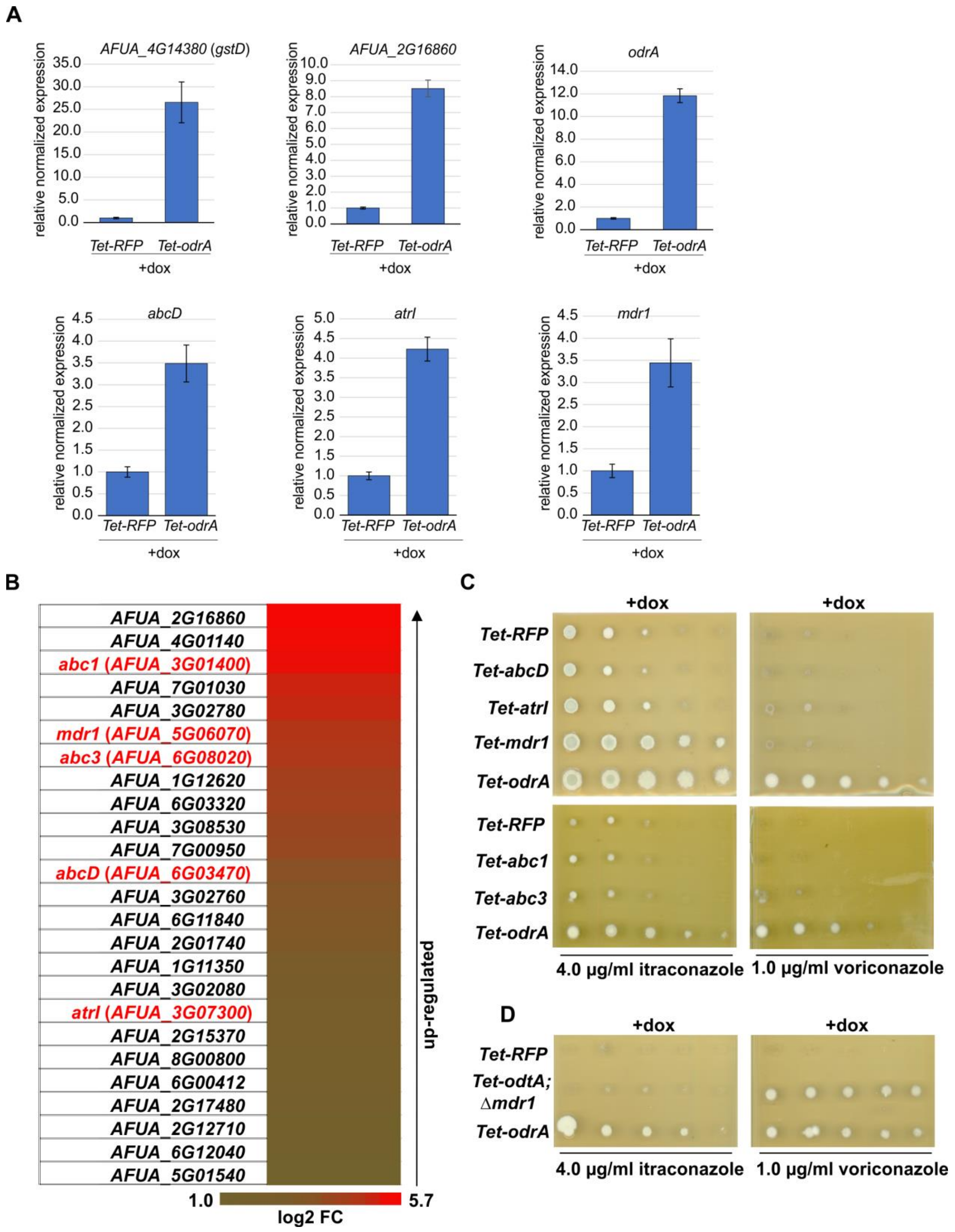

S7 Fig: Validation of the *odrA/mdu2* RNA-seq data and investigation of different transporter genes on drug resistance. qPCR experiments of *Tet-RFP* and *Tet-odrA*. Strains were incubated for 18h in liquid MM and shifted to fresh medium in presence (+dox) and absence (-dox) of 50 µg/ml doxycycline for additional 4h. *H2A* and *eIF2B* were used for normalization. Levels for the *Tet-RFP* strain (*Tet-RFP*) were set to 1. Graph indicates mean ± standard errors from two independent experiments each with three technical replicates. The relative expression levels of the genes *AFUA_4G14380*, *AFUA_2G16860*, *odrA/mdu2*, *abcD*, *atrI* and *mdr1* were measured. (B) Heat map of genes encoding transporters, which are up-regulated in the *Tet-odtA* strain in presence of doxycycline. Five of the up-regulated genes encode the multidrug transporters Mdr1, AbcD, Abc1, Abc3, and AtrI (highlighted in red). (C) Dilution spot-test of strains overexpressing the transporter encoding genes *abcD*, *mdr1*, *atrI,*

abc1 or *abc3*. Overexpression of *mdr1* increases the tolerance only to itraconazole, whereas the overexpression of *abc3* only weakly induces the tolerance to voriconazole. None of the other strains show a better growth on medium containing voriconazole or itraconazole in comparison to the control. (D) Dilution spot-tests of a *Tet-odrA; ∆mdr1* strain. The starting amount for the spot-tests were $1.5x10^5$ spores. 1/10 dilution steps were carried out. Spores were spotted on minimal medium supplemented with the azoles itraconazole or voriconazole. 50 µg/ml doxycycline was added for induction (+dox). Plates were incubated for three days at 37°C. As reference the *Tet-RFP* strain was used.
