# Supplementary material for: Induction of Aspergillus fumigatus zinc cluster transcription factor OdrA/Mdu2 provides combined cellular responses for oxidative stress protection and multiple antifungal drug resistance
Source: mBio. 2023 Nov 20;14(6):e02628-23. doi: 10.1128/mbio.02628-23 (PMC10746196; doi:10.1128/mbio.02628-23)
Supplement: Fig. S8 — OdrA binds to the promoter of mdr1 in a Tet-odrA-GFP strain. [file mbio.02628-23-s0008.pdf]

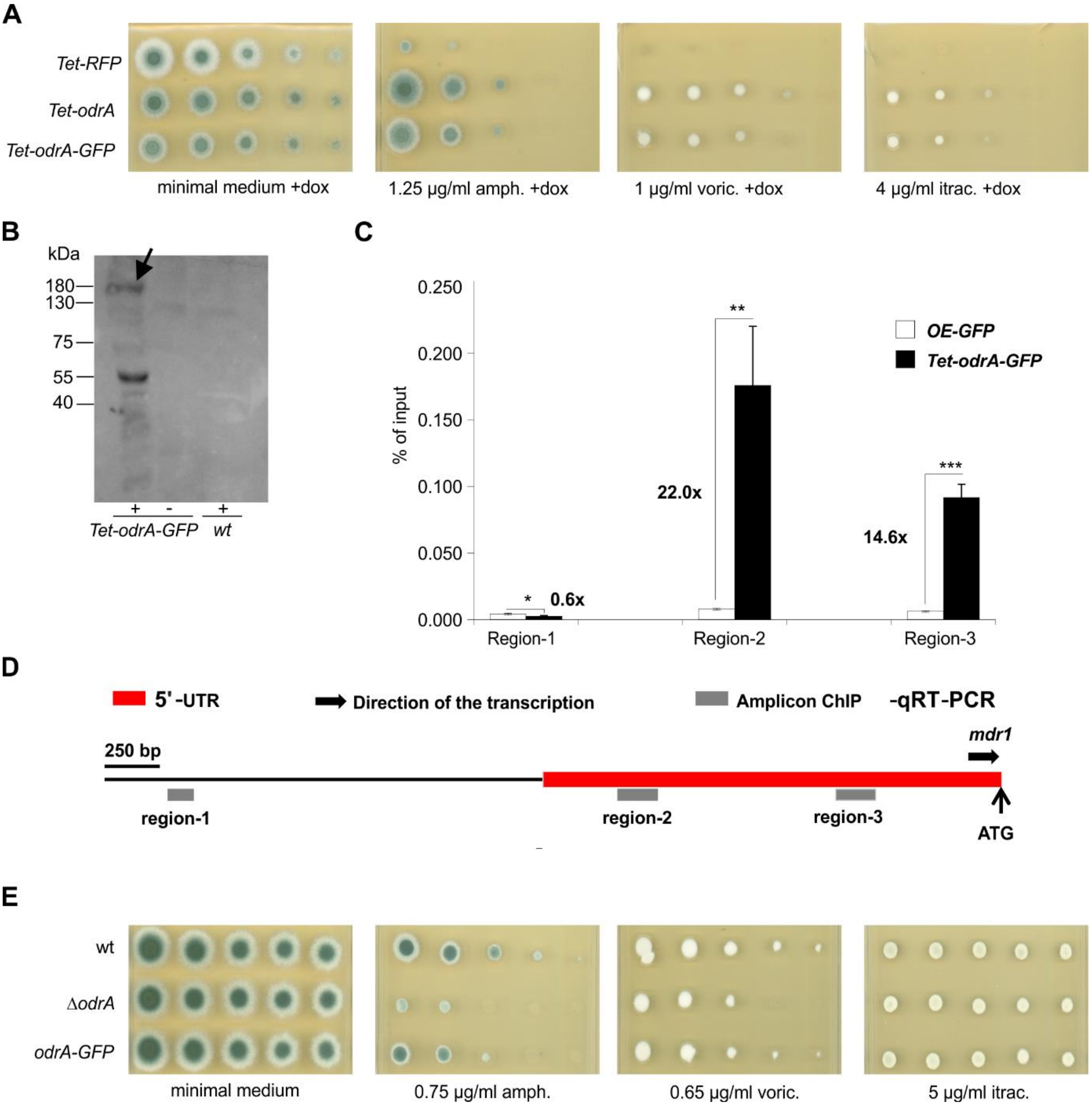

S8 Fig: OdrA binds to the promoter of *mdr1* in a *Tet-odrA-GFP* strain. (A) Dilution spot-test of *Tet-odrA-GFP* strain in comparison to *Tet-RFP* and *Tet-odrA*. Strains were diluted in 1/10 steps starting with 1.5 x10^5 spores. Spotting was carried out on minimal medium (MM) containing voriconazole, itraconazole or amphotericin B. All plates contained 50 µg/ml doxycycline for induction. Strains were grown for two (minimal medium with doxycycline but without drugs) or three days at 37°C. (B) Western experiments with the protein crude extract of the *Tet-odrA-GFP* strain. The strain was grown over-night in liquid MM for 18h. Received mycelium was shifted to fresh medium with (+) and without doxycycline (-) for additional 4h. As negative control the AfS35 wildtype was used in presence of doxycycline. A GFP antibody was used for detection. The calculated size of the OdrA-GFP fusion protein was 118 kDa. A signal could only be observed at 130 kDa (which might results from modifications of the protein) in the sample of the *Tet-odrA-GFP* strain in presence of doxycycline, indicated by the black arrow. (C) ChIP-seq experiments followed by qPCRs to identify the binding region of OdrA/Mdu2 to the promoter of *mdr1* using the *Tet-odtA-GFP* strain. *GFP* overexpression strain *OE-GFP* served as negative control. Strains were incubated in liquid minimal medium for 18h. Afterwards, doxycycline was added to a final concentration of 50 µg/ml for additional 4h. Three biological replicates for each strain were prepared. For analysis of the 5'UTR/promoter region of *mdr1* the primer pairs MB516_Fw/ MB517_Rv (Set-1), MB502_Fw/ MB503_Rv (Set-2), and MB504_Fw/ MB505_Rv (Set-3) were used. (D) Scheme of the *mdr1* promoter region. The amplifications of region-1, region-2 and region-3 are labelled in grey. The diagram shows an enrichment of the amplified region-2 and region-3, but not region-1 in comparison to

the control. (E) Spot-test *odrA-GFP*. The *odrA/mdu2* deletion strain and the wildtype (wt) were used as control. Strains were diluted in 1/10 steps starting with 1.5 x10^5 spores. Spotting was carried out on minimal medium (MM) containing voriconazole, itraconazole or amphotericin B. Strains were grown for two (minimal medium without drugs) or three days at 37°C.
