# Supplementary material for: Induction of Aspergillus fumigatus zinc cluster transcription factor OdrA/Mdu2 provides combined cellular responses for oxidative stress protection and multiple antifungal drug resistance
Source: mBio. 2023 Nov 20;14(6):e02628-23. doi: 10.1128/mbio.02628-23 (PMC10746196; doi:10.1128/mbio.02628-23)
Supplement: Fig. S9 — Influence of increased nuclear accumulation of OdrA/Mdu2 on drug response. [file mbio.02628-23-s0009.pdf]

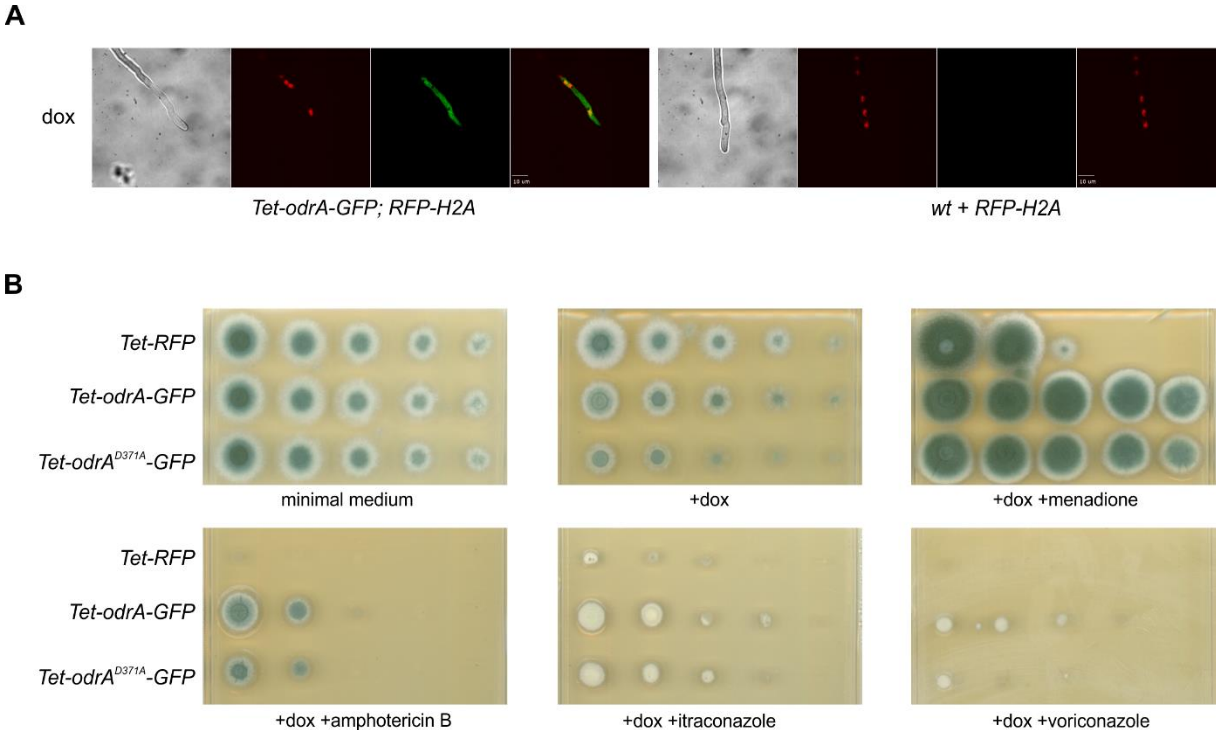

S9 Fig: Influence of increased nuclear accumulation of OdrA/Mdu2 on drug response. (A) Microscopy of the *Tet-odrA*^{D317A}-*GFP* strain and the wildtype. Strains were grown in minimal medium containing 5 µg/ml doxycycline overnight. Nuclei were visualized by an RFP tagged H2A protein in the corresponding strains. (B) Dilution spot-test of the *Tet-odrA*^{D317A}-*GFP* in presence 50 µg/ml doxycycline. dox and the *Tet-odrA-GFP* and the *Tet-RFP* and *Tet-odrA*. Strains were diluted in 1/10 steps starting with 1.5 x10^{5} spores. Spotting was carried out on minimal medium (MM) containing voriconazole, itraconazole or amphotericin B. All plates contained 50 µg/ml doxycycline for induction. Strains were grown for two (minimal medium with doxycycline but without drugs) or three days at 37°C. Minimal medium without doxycycline or antifungal drugs was used control. The combination of overexpression and mutation at position D371A in OdrA/Mdu2 does not increase the resistance in comparison to the *Tet-odrA* overexpression strain without point mutation. Resistance is even further reduced because the mutant overexpression strain has a more severe growth defect than the overexpression strain without mutation.
